# A numerical simulation approach for inflatable asymmetric geometries of orthotropic fabrics

**DOI:** 10.1038/s41598-026-40016-5

**Published:** 2026-03-10

**Authors:** Amir Samir Azer Abdelmaseeh, Adel Elsabbagh, Amr Yehia Elbanhawy

**Affiliations:** 1 Mechanical Design and Production Department, Faculty of Engineering, Ain Shams University, Cairo, 11517 Egypt; 2 Mechanical Power Department, Faculty of Engineering, Ain Shams University, Cairo, 11517 Egypt; 3 Center for Vibration, Sound and Smart Structures, Faculty of Engineering, Ain Shams University, Cairo, 11517 Egypt; 4 Energy Technology and Climate Change Laboratory, Faculty of Engineering, Ain Shams University, Cairo, 11517 Egypt

**Keywords:** Inflatable structure, Inflatables’ FEA simulation, Inflated wind turbine blade, PVC-coated fabrics, Energy science and technology, Mechanical engineering, Composites

## Abstract

Inflatable structures are lightweight, easily transported, manufactured, and deployed. These advantages make them widely applicable in civil, aerospace, and recently in wind energy applications. They are also characterized by large deviations between the un-inflated and inflated geometries, making the design process of a structure with well specified target geometry under different loading conditions very challenging. This paper presents a comprehensive framework to predict the geometry of polyvinyl chloride coated polyester fabrics and their behavior under loads. The presented framework includes detailed characterization of the material properties to be fed to commercially available software for FEA of inflatable structures. The developed model is then used to predict the ability of the structure to withstand loads without failure or wrinkling. The framework is validated against 3D scanning of the inflated geometry of four different cases which are chosen to represent different levels of complexity including weld anisotropy, geometric nonlinearity in asymmetric forms, and the effect of internal reinforcements. Comparisons between numerical predictions and experimental outcomes demonstrate the framework’s ability to capture deformation and strain behavior for complex inflatable structures. The proposed workflow provides designers with a valuable tool to design and build inflatables with a higher level of dimensional accuracy and load-bearing capacity, enabling applications beyond current limitations.

**Supplementary Information:**

The online version contains supplementary material available at 10.1038/s41598-026-40016-5.

## 1. Introduction

Inflatable structures are characterized by their lightweight nature, ease of fabrication, and rapid deployment making them suitable for diverse applications in aerospace, civil engineering, and architectural design. Advances in the development of such structures broadly rely on progress in three domains: materials, design-for-manufacturing, and high-fidelity modeling and simulation, since current simulation approaches need to address several unresolved challenges in simulating free-form non-tubular, and asymmetric inflatable geometries.

Structurally, inflatable membranes are typically fabric composites, combining high-strength textiles such as polyester or glass fiber with impermeable coatings like polyvinyl chloride (PVC), nitrile butadiene rubber (NBR), Polychloroprene elastomer (CR), Silicon, and butyl rubber. Those coatings are used to maintain internal pressure^1^. Textile composite types and mechanical behavior are briefly described by Oñate and Kröplin^2^. These composites exhibit nonlinear and anisotropic behavior due to their woven architecture and coating layers, requiring biaxial testing to capture properties essential for accurate finite element analysis (FEA). The work by Galliot et al.^3^ quantified how warp-fill yarn interactions govern PVC fabric mechanics, while the creep loading behavior of PVC fabrics is presented by Żerdzicki and Jacomini^4^. Several studies highlighted the influence of yarn interaction and fabrication techniques on mechanical performance, necessitating experimental validation for specific materials, such as PVC-coated polyester or ETFE foils^5,6^.

A survey of the literature shows an imminent need for a robust simulation tool, validated through geometry and mechanical load testing, to support the design of innovative inflatables replacing conventional structures in more engineering applications. Models of inflatable structures have predominantly employed the Uniform Pressure Method (UPM) for numerical simulations as demonstrated by Schwoll^7^, with limited adoption of the more sophisticated dynamic explicit Coupled Eulerian-Lagrangian (CEL) technique^8^. While these methods have provided valuable insights, a critical gap remains as neither approach has undergone comprehensive experimental validation, raising questions about their applicability under real manufacturing conditions. Moreover, most of the work particularly focused on simple geometric configurations, with cylindrical beams and axisymmetric structures representing the majority of studied cases. This limited focus has left a significant knowledge gap regarding the predictive capabilities for complex, non-symmetric geometries that are increasingly relevant in advanced engineering applications.

Another important aspect of inflatables modeling is how material is represented. Inflatable fabrics are usually described as a hyperelastic non-linear orthotropic material as is in Jekel et al.^9^. However, the accuracy of capturing the mechanical properties of the woven fabrics in FEA models is challenging due to the complex stress-strain relationships inherent in these materials. To address these complexities, user-defined material subroutines such as VUMAT (used within Abaqus/Explicit) have become essential tools for implementing advanced constitutive models. The development of a material model implemented through the VUMAT interface (HypoDrape) for simulating fabric drape and forming was presented by Thompson et al.^10^. The inflation process can be considered as a shell forming process, in which internal pressure induces large deformations and strains on the geometry. Unlike traditional forming methods, the deformation in inflatable structures is fully or partially reversible, allowing the structure to return to its original configuration upon deflation.

The geometrical behavior of the free form inflated structures, and the manufacturing methods of those structures has less focus within the recent work. Beams, arches, spheres and 2D inflated geometries have been thoroughly investigated. However, FEA models of other arbitrary shapes were not equally considered. Even for the few developed models, too many limitations exist. Moreover, there is a lack of experimental validation of FEA models for arbitrary shaped inflatables. To exemplify, a three-dimensional nonlinear (FE) model for inflated beams was proposed by Apedo et al.^11^​ based on a Timoshenko beam model with homogeneous orthotropic fabric and derived using the total Lagrangian form of the virtual work principle. This model is limited to circular beam cross-sections, and to linearly elastic orthotropic fabrics. Lampani et al.^12^ utilized FEA for modeling inflatable structures but again addressed simple geometries without delving into complex free-form shapes. Particularly, the study considered the inflated materials to be isotropic and represented as shell elements. A dynamic explicit approach was used with employment of an Arbitrary Lagrangian-Eulerian (ALE) method to discretize the internal gas of the airbag using three dimensional elements, enabling coupled fluid-structure interaction analysis during inflation.

Performance of inflatable fabric-based structures has been previously addressed with focus on the ability of simple structures to withstand loads, and/or the effect of inflation pressure on the stiffness of the structure. A theoretical FE model for the analysis of the axisymmetric inflatable beams (straight and tapered) was developed by Elsabbagh^13^ and the model was utilized to predict the effect of the internal pressure on the wrinkling loads. However, this model also lacks experimental validation. Bending and buckling behaviors of thin-walled cylindrical inflatable beams was theoretically presented by Le Van et al.^14^ and Nguyen et al.^15^ while the deformation of the inflated tapered cantilever beam under concentrated loads was introduced by Chen et al.^16^. The mechanical behavior of highly pressurized fabric tubes subjected to bending loads was addressed by Thomas et al.^17^. Coupled bending and wrinkling behavior of simply supported inflated beams was studied by Ye et al.^18^ and He et al.^19^. through experimental and theoretical approaches.

Despite progress in both simulation methods and material models, a critical gap remains. There is currently no validated high-fidelity FEA framework capable of predicting both the geometry and mechanical performance of complex, free-form inflatable structures. The three main observed limitations in the literature can be summarized as follows: (1) focus on simple structures such as beams; (2) lack of experimental validation in predicting complex shaped inflatables; and (3) simplified material models that may not adequately capture the mechanical behavior of inflated complex structures under loads.

To address these gaps, this study presents a comprehensive simulation framework that integrates a nonlinear anisotropic material model, implemented through a VUMAT subroutine, into a dynamic FEA environment for the inflation of arbitrary shapes. A two-phase validation strategy is employed: (i) verification of predicted geometries against 3D photogrammetry of physical prototypes, and (ii) assessment of mechanical performance through comparison with analytical solutions for inflated beams and the free-form geometries. To demonstrate the framework’s capabilities, four representative cases are investigated: a double-layer welded pillow, a stiffened cuboid, a twisted lofted shape, and its stiffened variant. These examples collectively address three unresolved challenges: weld anisotropy in multi-layer assemblies (examining interlayer shear and stiffener junction behavior), geometric nonlinearity in asymmetric forms (quantifying twist-induced wrinkling), and load-path complexities introduced by internal reinforcements. By moving beyond simplified geometries and providing systematic experimental validation, this work establishes a practical and reliable tool for the design and analysis of complex inflatable structures. The outcomes are directly relevant to advanced applications, including architectural membranes and renewable energy systems, such as the inflatable wind turbine rotor blades being developed in an undergoing project at Ain Shams University^20^.

## 2. Methodology

### 2.1. Proposed framework

**Fig. 1:**
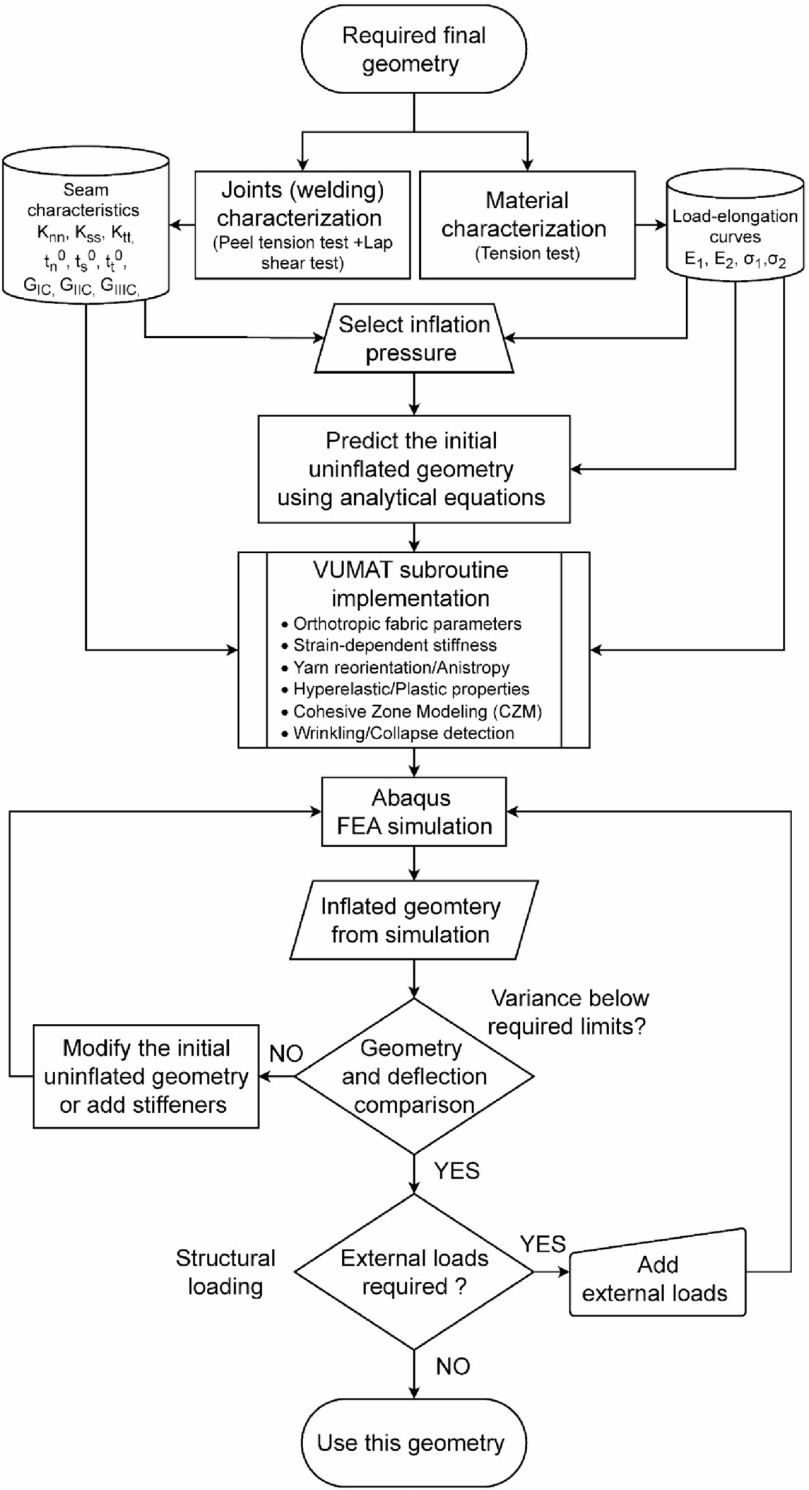
Proposed flow chart for inflatable geometry determination.

The framework presents a systematic approach for designing inflatable structures with customizable geometry and structural properties, based on material selection, inflation pressure, and structural analysis. As illustrated in (Fig. 1), the process begins by defining the target geometry of the structure, followed by selecting a suitable material according to its mechanical properties. Material characterization tests are then performed on the selected material, including evaluations of welding joint properties. These tests provide key parameters such as the load-elongation curve, elastic moduli (E_1_ and E_2_), and ultimate tensile strength which define the maximum allowable tensile forces under internal pressurization.

Once the material is characterized, an appropriate inflation pressure is determined based on its strength in addition to the seam strength. This pressure is sufficient in cases where no external loads are applied, and lower stiffness is acceptable. The initial inflated geometry is then estimated using thin-walled analytical equations, where strain is predicted from the material’s load-elongation curve at the calculated stress.

Next, finite element analysis (FEA) simulation is subsequently performed via Abaqus/CAE software (versions 2020 and 2022)^21^ to predict the final geometry. The resulting geometry is compared to the target specifications via ZEISS INSPECT geometry comparison software (2023 release), previously known as (GOM Inspect)^22^. If deviations exceed acceptable tolerances, the initial geometry is refined through adjustments or the addition of internal stiffeners.

If the variance does not meet the requirements, the process is repeated from the FEA stage. Upon achieving an acceptable geometry, external loads (if relevant) are applied in the FEA model to evaluate structural performance under operational conditions. This step may lead to further geometry modifications or reinforcement with additional stiffeners.

Finally, if the structure meets all design requirements without the need for further modifications, the finalized geometry is approved for practical implementation.

### 2.2. Material characterization

**Fig. 2:**
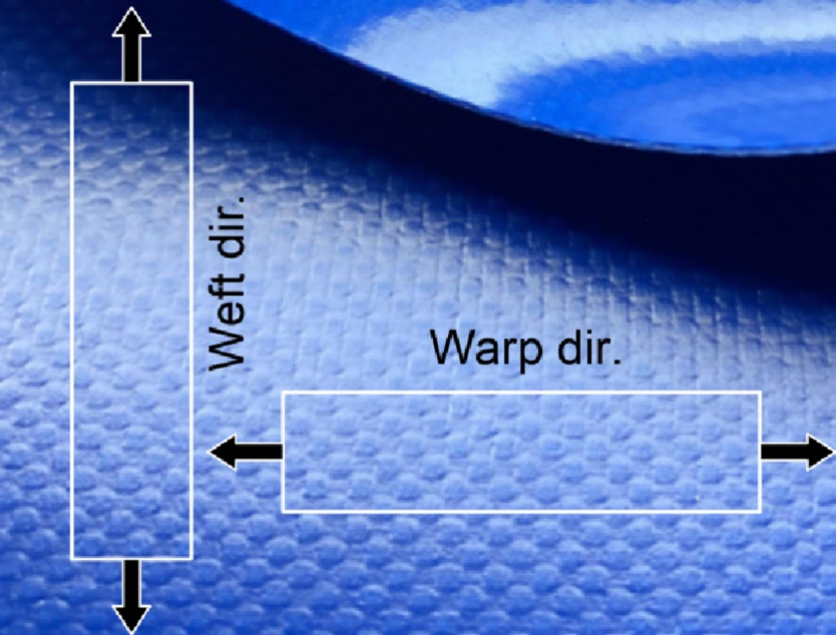
Warp and Weft directions for the chosen PVC-coated fabrics.

**Fig. 3:**
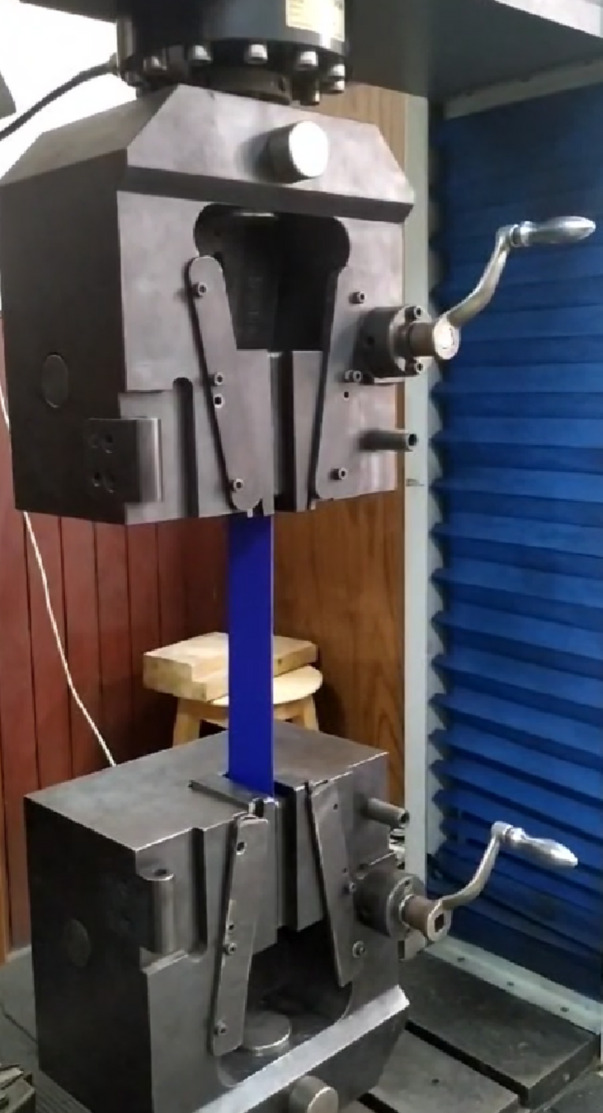
Biaxial tension test for the PVC fabrics.

To confidently validate the developed simulation models with experimental results, the model should be provided with accurate characterization of the materials under consideration. In all the experimental work in this paper, PVC-coated fabrics were used as membrane materials for the inflated structures and the internal stiffeners. These fabrics exhibit anisotropic mechanical behavior, with distinct properties in the warp and weft directions. Accordingly, an orthotropic material model was adopted to capture the directional elastic behavior, as illustrated in (Fig. 2). The tensile strength and elasticity moduli in both directions are obtained experimentally via uniaxial tension tests (Fig. 3) according to ISO 1421:2016^23^ on a universal testing machine with a capacity of 300kN. Strip test method is selected where the full width of the sample is gripped in the jaws, and the test was conducted at a constant rate of extension equal to 100 mm/min. Specimens were cut along the desired fabric orientations, with dimensions of 50 mm in width and 200 mm in nominal length.

**Fig. 4:**
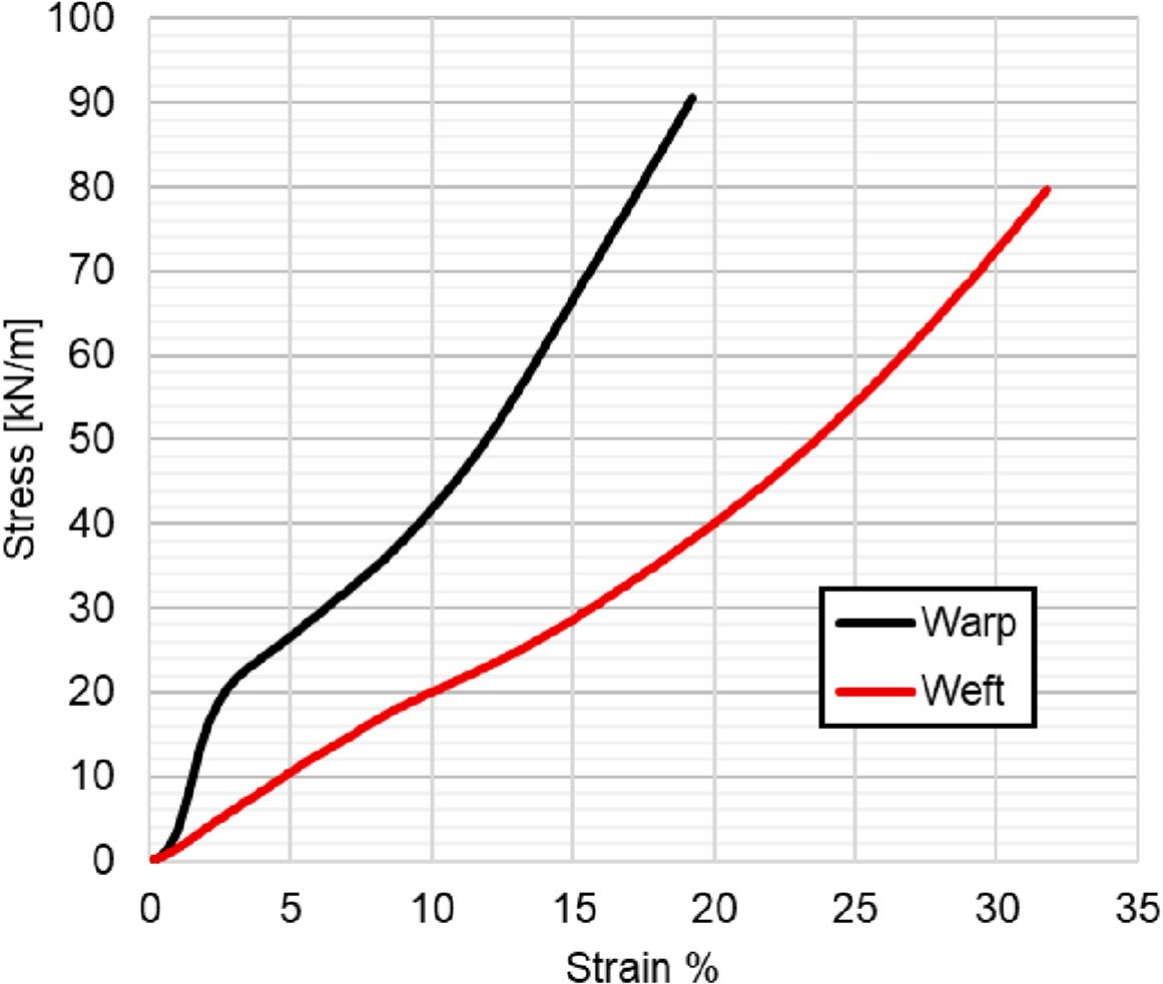
Stress-strain curves for PVC coated fabrics plotted via uniaxial tensile tests.

The results of the tension tests are summarized in (Table 1), while the stress-strain curves for both directions are shown in (Fig. 4). These curves illustrate the material’s response under uniaxial loading, highlighting its hyperelastic behavior and ultimate tensile strength.

**Table 1:** PVC samples’ specifications and results of mechanical testing.

Fabric direction	Warp direction	Weft direction
Elastic modulus	E_1_=480 MPa	E_2_=358 MPa
Force at break	2.9 KN	2.55 KN
Ultimate strength	90.625 MPa	79.688 MPa
Elongation at break	38.76/200 = 19.38%	63.6/200 = 31.8%
Poisson’s ratio (variable)*	0.499 ~ 0.437	0.499 ~ 0.355
**Samples specifications** • PVC fabric (polyester with and PVC covering from each side) • Nonlinear hyperelastic orthotropic material • Length = 200 mm • Thickness = 0.64 mm • Density = 1.2 gm/cm^3^ • Temperature resistance [−30 °C to + 70 °C] *Poisson’s ratio is calculated as a function of the strain via (Eq. 1)^24^

1 $$\:\nu\:=\:\frac{1}{\varepsilon\:}\left(1-\frac{1}{\sqrt{1+\varepsilon\:}}\right)\:\:\:\:\:\:\:\:$$

The fabric exhibits linear orthotropic behavior up to tensile loads of approximately 20 MPa in the warp direction and 15 MPa in the weft direction. Beyond these limits, nonlinear effects arise due to the plasticity of the PVC coating. This transition must be incorporated into the FEA model to ensure accurate prediction of large deformations.

### 2.3. Seam characterization

**Fig. 5:**
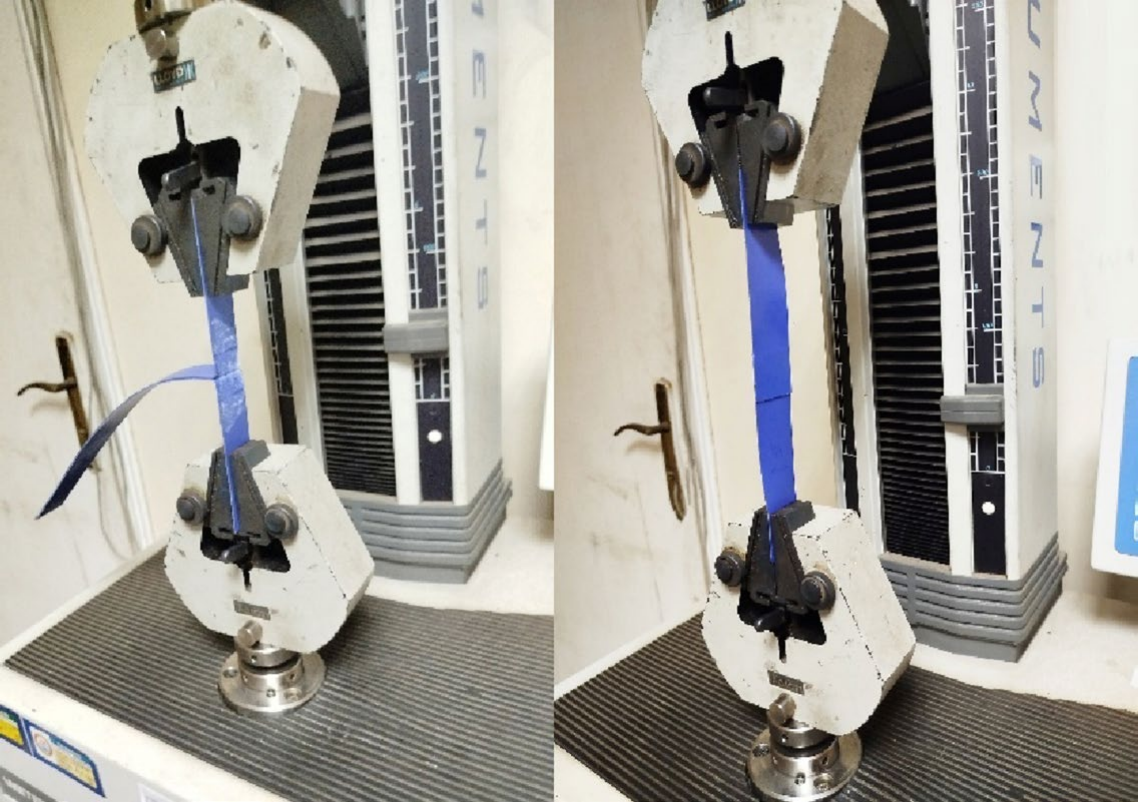
Peel test on welded joint (left) and lap shear test (right).

Welded samples were subjected to peel resistance test^25^ and shear adhesion test^26^ (Fig. 5) according to ASTM D1876-08(2023) for adhesive added and hot air welded joints^27^ and ASTM D751-06(2017)^28^. The primary objective of these tests is to determine the cohesive element parameters for the Abaqus model, as summarized in (Table 2).

**Table 2:** Summary of CZM parameters, experimental determination tests and calculation methods.

CZM parameter	Physical meaning	Experimental test	Calculation method	Value obtained
$\:Knn$	Normal Stiffness	T-Peel test	Slope of the initial linear elastic part of the force-displacement curve (ΔF/Δδ), divided by the initial area	687 KPa/mm = 0.687 N/mm^3^
$\:{{t}_{n}}^{0}$	Normal Strength	T-Peel test	Peak force from the test, divided by the initial cross-sectional area of the seam	10 MPa
$\:{G}_{IC}$	Mode I Fracture Energy	T-Peel test	Area under the force-displacement curve (until complete failure), divided by the final torn area. This area represents the total energy absorbed during failure	0.08 N/mm
$\:Kss,Ktt$	Shear Stiffness	Lap shear test	Slope of the initial linear elastic part of the force-displacement curve, divided by the initial area	261, 195 KPa/mm
$\:{{t}_{s}}^{0},{{t}_{t}}^{0}$	Shear Strength	Lap shear test	Peak force from the test, divided by the initial cross-sectional area of the seam	9, 7 MPa
$\:{G}_{IIC},{G}_{IIIC}$	Mode II/III Fracture Energy	Lap shear test	Area under the force-displacement curve (until complete failure), divided by the final torn area	0.4, 0.25 N/mm

**Fig. 6:**
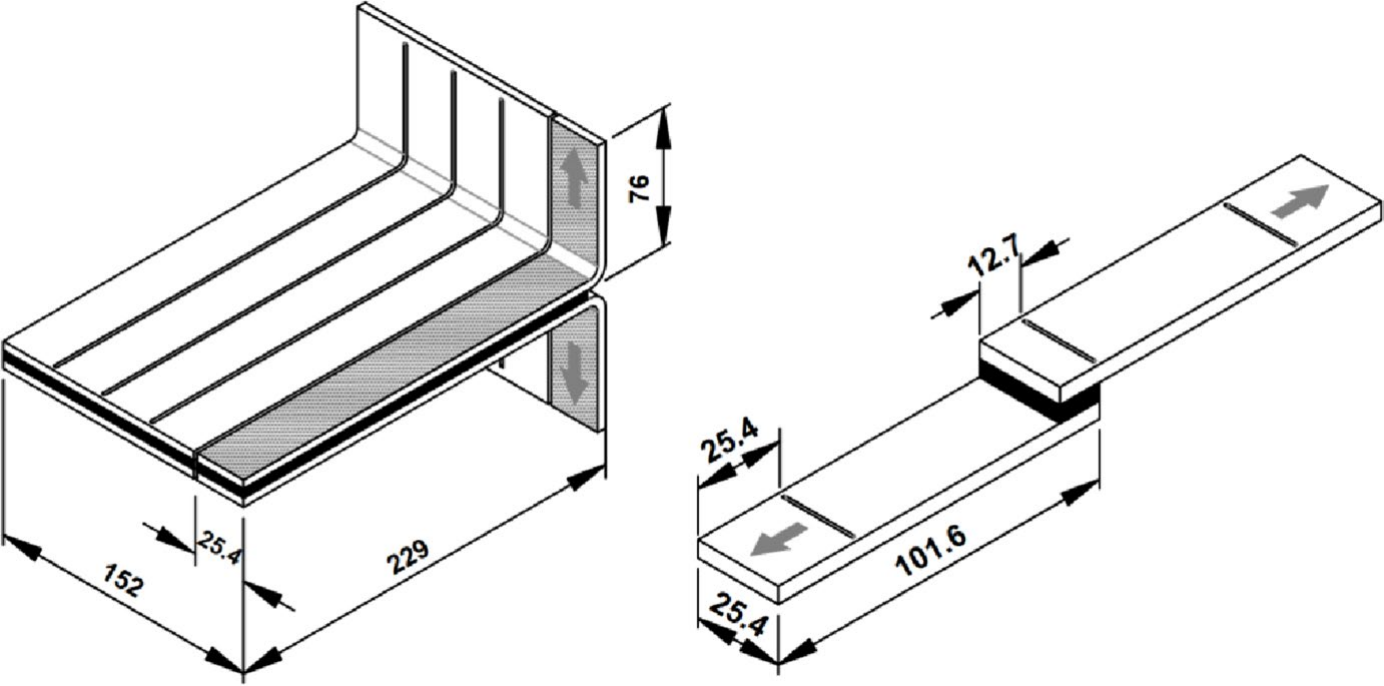
Dimensions of peel test panel (left) and lap shear test specimen (right).

T-peel tests were performed on specimens of 25 mm (1 in.) wide, cut from test panels measuring 152 mm (6 in.) in width and 305 mm (12 in.) in length, with bonded length of 241 mm (9 in.) and unbonded griping length of 76 mm (3 in.) as shown in (Fig. 6). The tests were carried out on a universal testing machine at a constant speed of 254 mm/min, and the resulting force-displacement curve is plotted then subsequently converted to traction–separation relationships which provided the necessary input parameters for the cohesive-zone model.

The elastic stiffness (K_nn_) was calculated from the initial slope (ΔF/Δδ) of the curve, where the initial area of the seam is equal to (25.4 × 0.06 mm^2^) where 0.06 is the thickness of the adhesive layer. The critical fracture energy (G_IC_) was calculated by computing the total area under the curve, which represents the work of separation, and dividing by the final torn area.

For shear testing, single lap welded joints were prepared with an overlap length of 12.7 mm and specimen dimensions of 25.4 mm width by 101.6 mm length. The bonded region was centered, while the free ends were clamped in tensile grips. Tests were conducted at a constant speed of 0.05 in./min (≈ 1.27 mm/min) until failure.

**Fig. 7:**
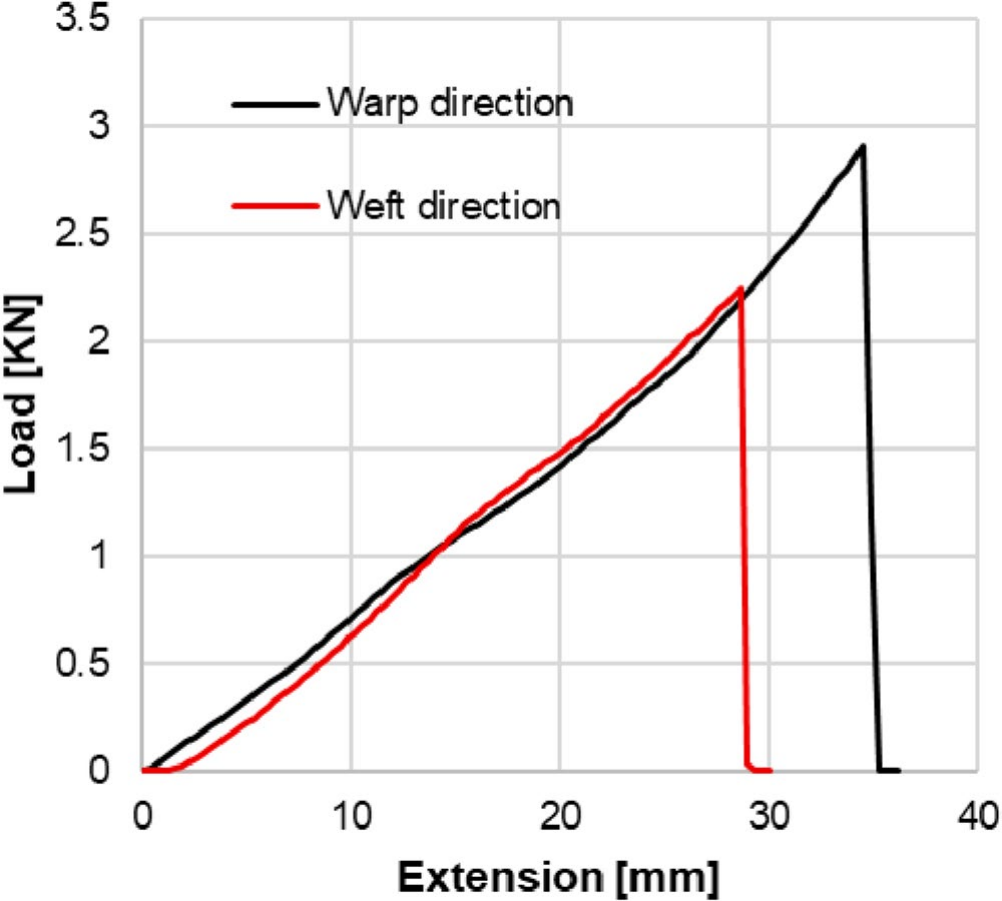
Load-extension data generated in the lap-shear test in both direction.

Load-displacement data were recorded and used to determine shear stiffness in both directions (Fig. 7) and the corresponding fracture energies.

### 2.4. Test cases’ preparation

This section outlines the design and manufacturing process of the test cases used for experimental validation. Four test cases with various levels of complexity were designed. The four chosen cases follow the following arrangement: (1) a simple 2D symmetric structure in the form of a pillow; (2) a 3D symmetric shape in the form of a cuboid with a middle inner stiffener; (3) a 3D asymmetric, more complex, twisted and lofted form; and (4) the same twisted shape with two inner stiffeners, where the material of the tension stiffeners inside the shape is also PVC-coated fabric, with welding methods described in the drawings. These four test cases were fabricated and inflated with air at 150 kPa (1.5 bar). The inflated geometry was scanned via 3D photogrammetry, and the outcomes of the 3D scanning were compared with the predictions of the developed framework; then, the amount of deviation was quantified. Represented as normal to the surface, the error is expressed as the difference between the FEA model and the geometry resulting from 3D scanning.

To physically build the inflated geometries, the PVC fabric, whose material properties were fully characterized in the previous section, was cut to size and joined together to build the intended structure. Fabric welding had a considerable effect on the behavior of the inflated structure. Hence, a detailed description of the fabric joining method was essential. In this study, the fabrics were joined together via two methods. The first joining method involved hot air seam welding using machine model number YC-RBW08, Yecheng Machinery^29^, at a temperature of 550 °C, welding speed of 0.4 m/min with no roller speed offset, and an airflow rate of 1.2 m³/min at a pressure of 10 kPa with a 45 mm² nozzle. The upper roller pressure was 420 kPa, the seam width was 30 mm, and polyurethane-coated rollers 80 mm in diameter were used. The second method of joining involved the use of ethyl 2-cyanoacrylate adhesive between the PVC layers.

**Fig. 8:**
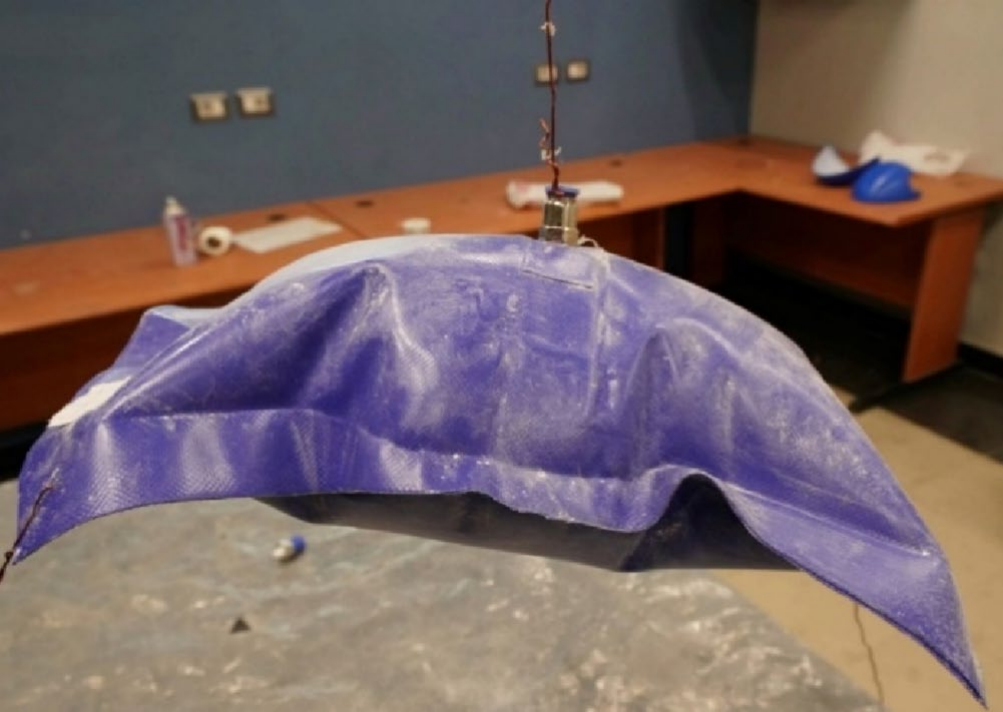
Inflated pillow.

Starting with the simplest form, the 2D membrane inflatable consists of two identical planar welded rectangular sheets with dimensions of 360 × 260 mm^2^ (Fig. 8).

**Fig. 9:**
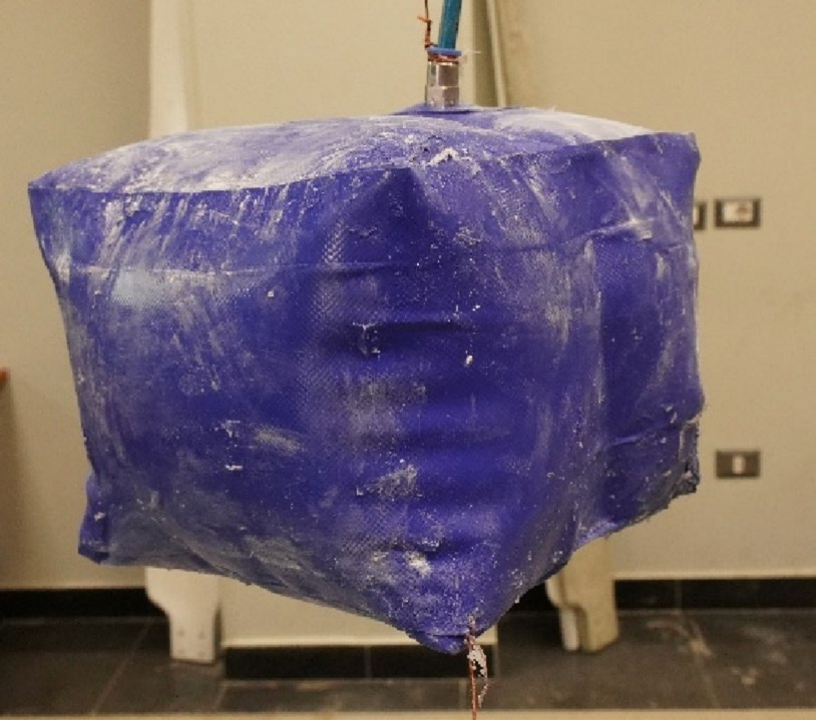
Inflated Cuboid with inner stiffener covered with talcum powder for scanning purposes.

**Fig. 10:**
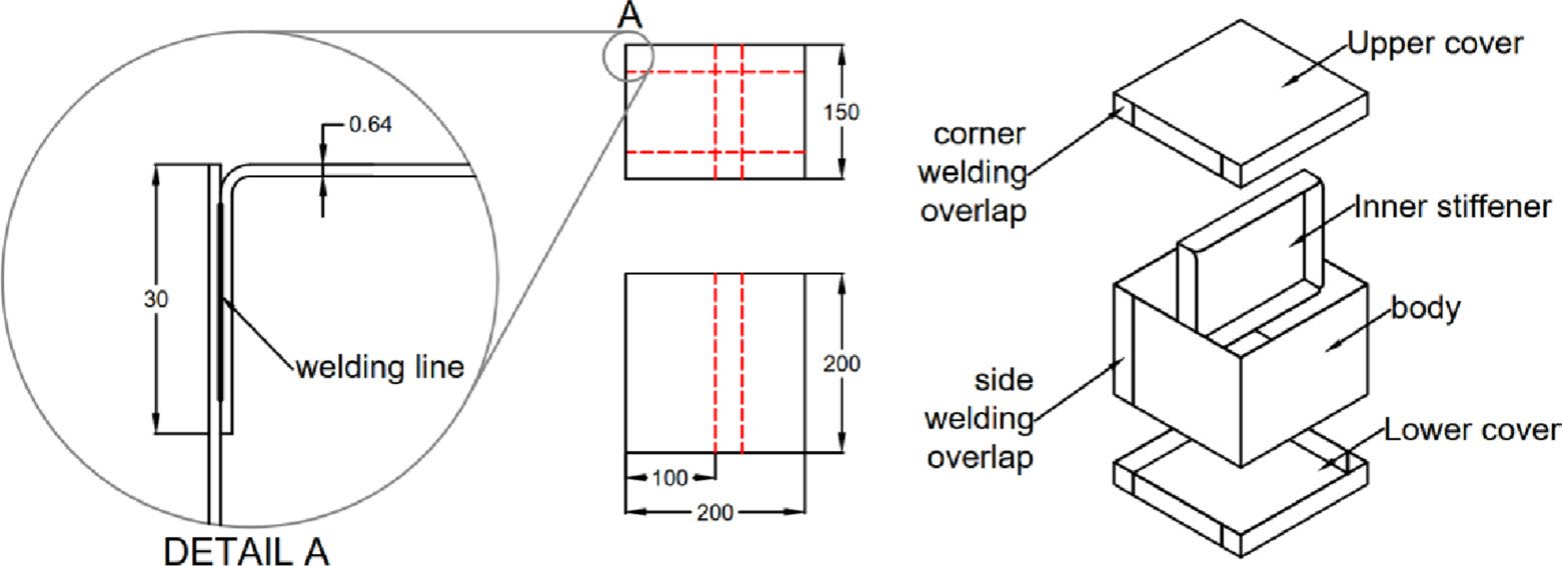
Dimensions and welding position for the cuboid with inner stiffener.

The second test case is an inflatable cuboid stiffened by a single internal stiffener in the middle, as shown in (Fig. 9). The main dimensions of the cuboid are 200 × 200 × 150 mm^3^ (Fig. 10). The figure also illustrates the details of the 30 mm internally welded hot-air joints. All dimensions indicated in the drawings are input dimensions.

The third test case is more complex, featuring an asymmetric lofted shape with a twist angle of 20° along its axis. (Fig. 13) shows the construction and main dimensions of this sample.

**Fig. 11:**
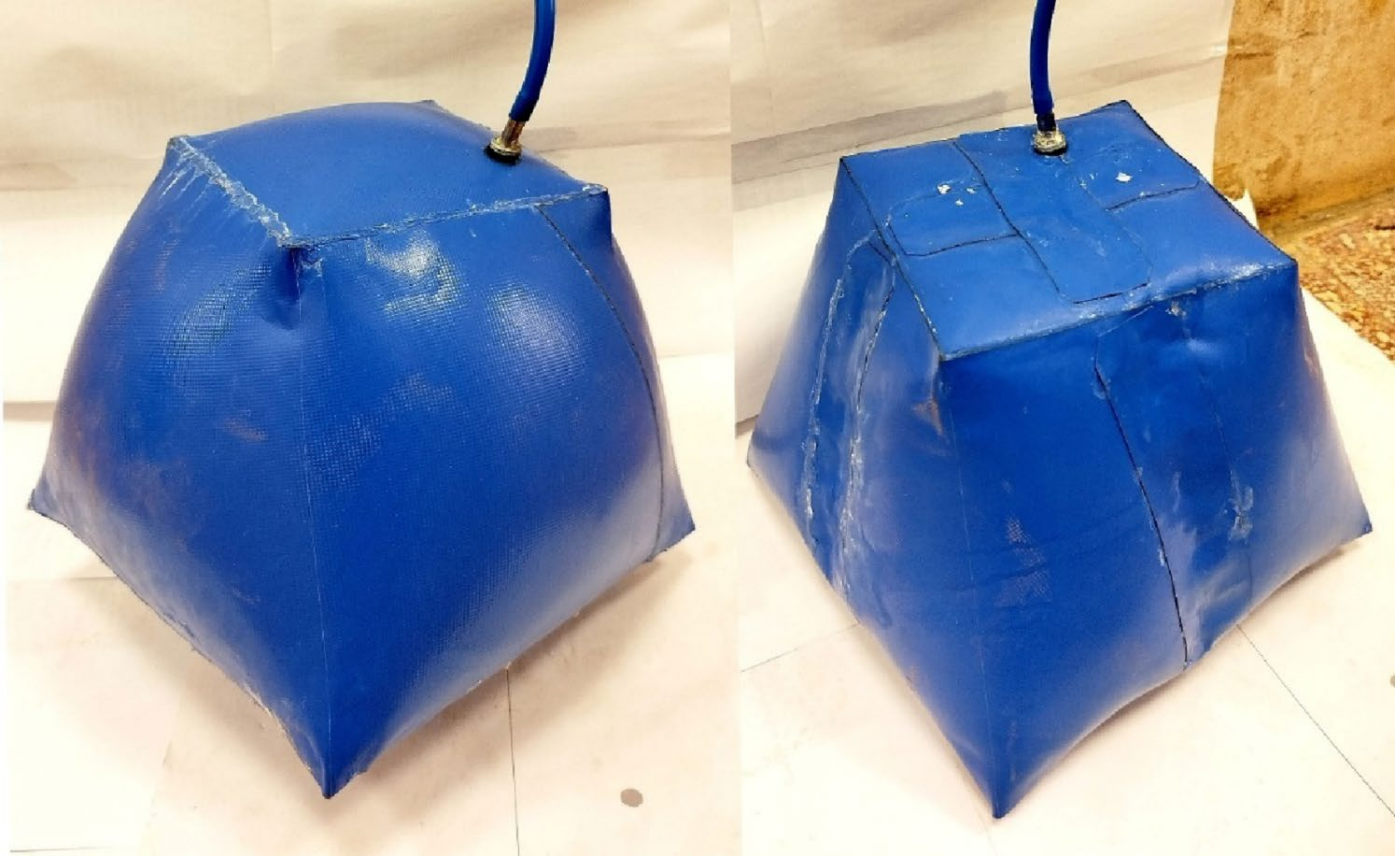
Asymmetric lofted shape with a twist angle equal to 20° along its axis without internal stiffeners (left) and with internal stiffeners (right).

**Fig. 12:**
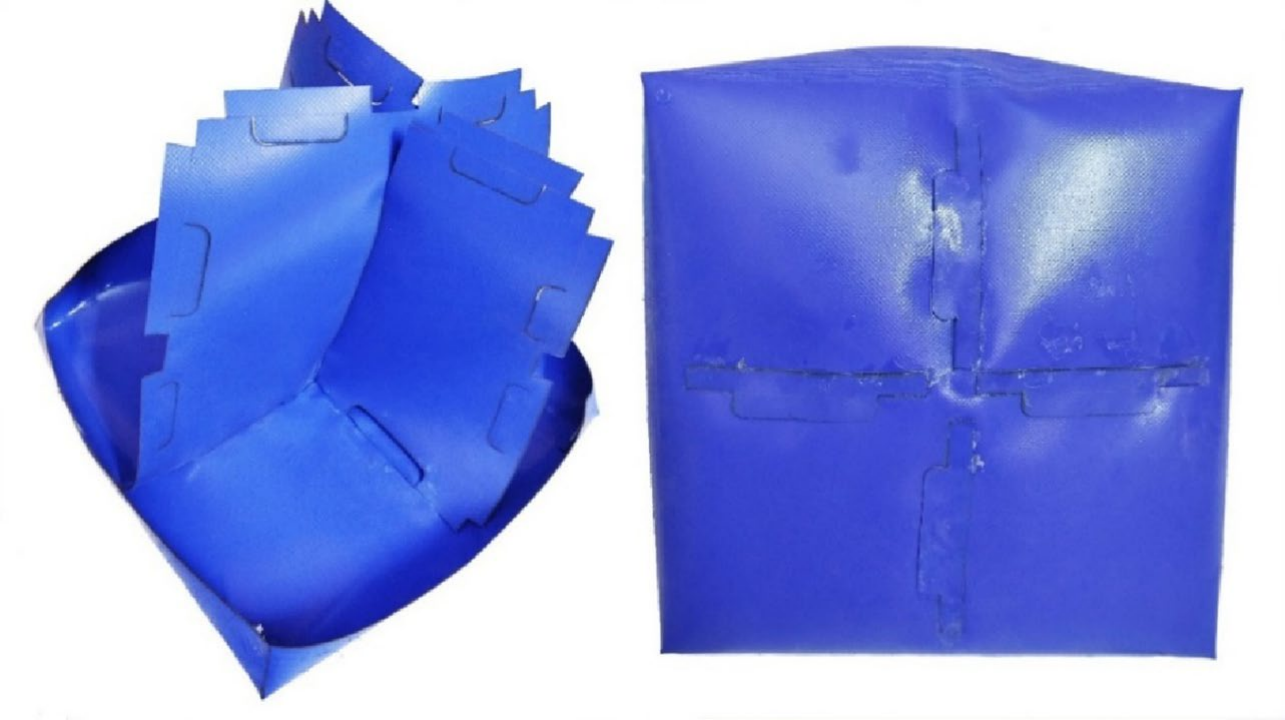
Adhesive shear resistance joint for the internal stiffeners.

**Fig. 13:**
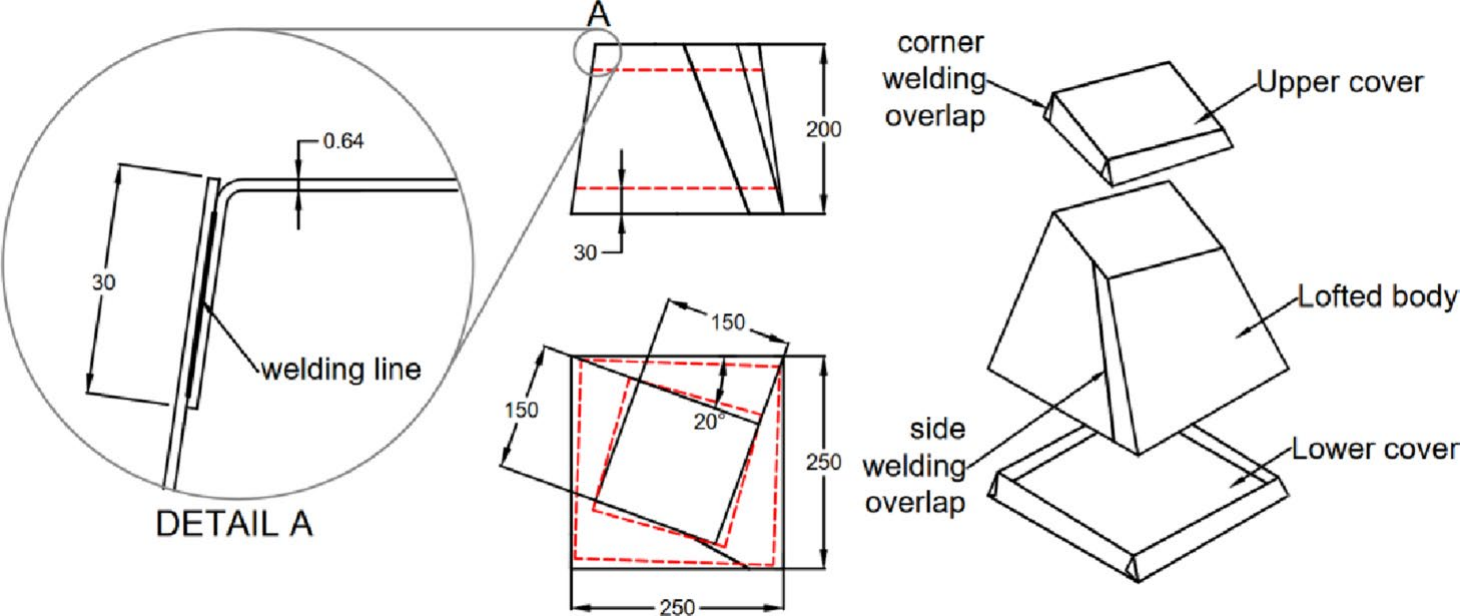
Dimensions and welding positions for the asymmetrically lofted shape without stiffeners.

**Fig. 14:**
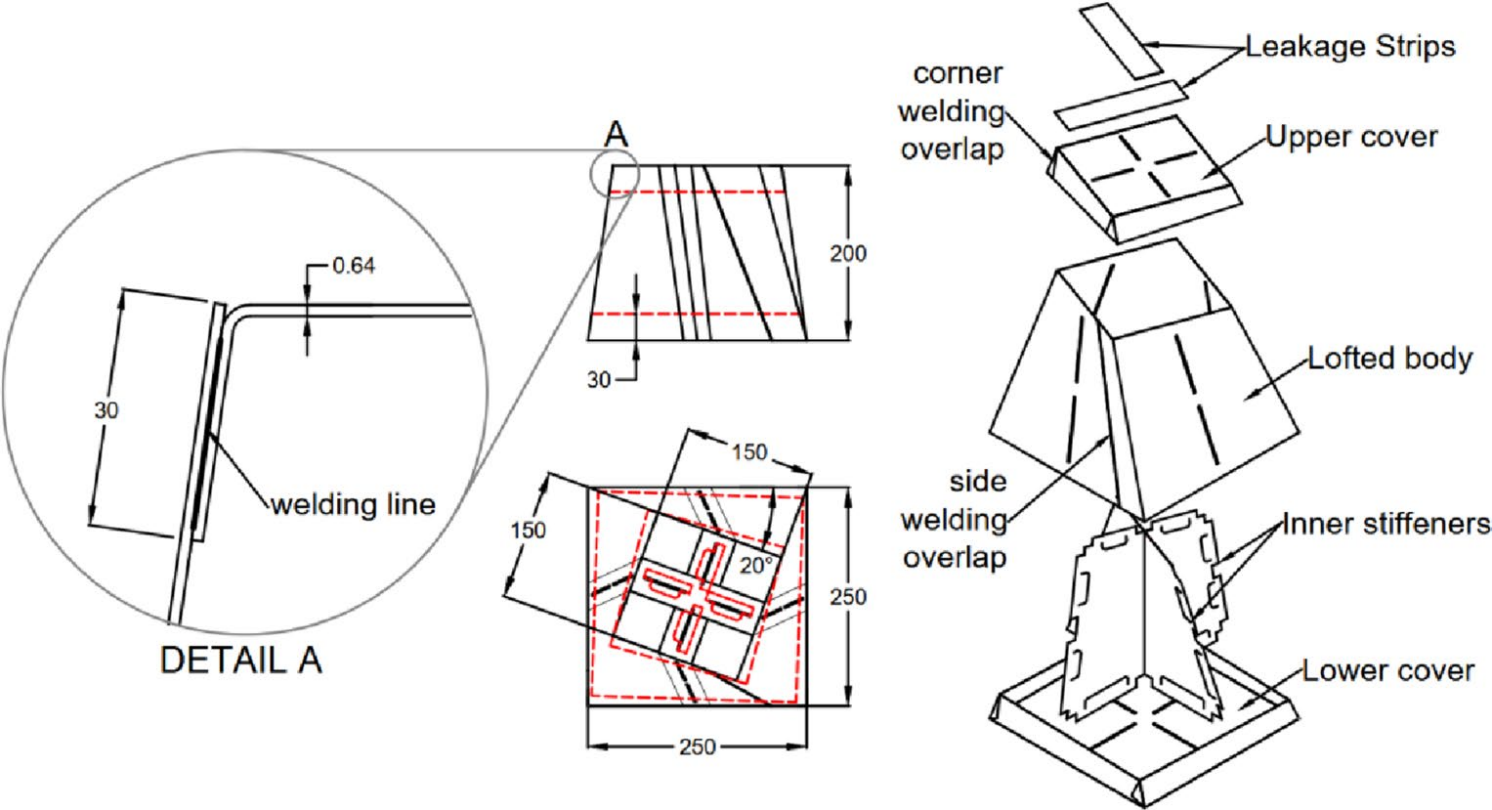
Dimensions and welding positions for the asymmetrically lofted shape with two inner stiffeners.

The fourth test case retains the same geometry as the third one but includes two internal stiffeners (Fig. 11). These stiffeners serve three key purposes: (1) maintaining the geometry without excessive deformation, (2) reducing untwisting effects resulting from inflation, and (3) demonstrating stress redistribution in inflatable structures with internal stiffeners. The stiffener assembly is shown in (Fig. 12) and is further described in patent PCT/EG2025/050007^20^. This configuration alters the stress distribution across welded joints by converting peel resistance forces into shear resistance, thereby improving joint integrity. (Fig. 13) and (Fig. 14) show the construction of test cases three and four.

### 2.5. Description of the model

#### 2.5.1. Nonlinear orthotropic FEA for free-form inflatables

This study employs dynamic explicit finite element analysis (Abaqus/Explicit 2020) via a Coupled Eulerian–Lagrangian (CEL) formulation to capture fluid–structure interactions between the inflatable membrane (Lagrangian domain) and internal air (Eulerian domain). The CEL approach addresses key limitations of uniform pressure methods by (1) modeling realistic air-structure coupling during inflation and deformation, and (2) capturing large deformations and wrinkling instabilities.

For fabric material modeling, a custom VUMAT subroutine replaces Abaqus’s native VFABRIC because of its critical shortcomings: VFABRIC assumes linear elasticity with a constant stiffness matrix, neglecting strain-dependent stiffness and yarn reorientation-induced anisotropy. Furthermore, its inability to model hyperelasticity or plasticity precludes the simulation of non-recoverable deformations, such as permanent wrinkling or fiber slippage.

A quick comparison between both subroutines is described in (Table 3).

**Table 3:** Comparison between the VFABRIC and VUMAT subroutines.

Subroutine	VFABRIC	VUMAT
Material Behavior	Orthotropic Linear Elasticity with no plasticity	Linear/nonlinear, anisotropic, hyperelastic with user-defined yield criteria
Strain rate effect	No	Yes
Key assumptions	1. Linear elasticity in warp/weft directions 2. Small strains (< 5%) 3. Plane stress (2D membrane) 4. No material nonlinearity	1. No inherent assumptions (user-controlled) 2. Can handle finite strains 3. Supports 3D solids, shells, membranes 4. Captures nonlinearity, plasticity, damage
Stiffness matrix	3 × 3 Matrix (2D plan stresses)	6 × 6 Matrix (full 3D)
	$$\:{C}_{VFABRIC}=\left[\begin{array}{ccc}\frac{{E}_{1}}{1-{\nu\:}_{12}{\nu\:}_{21}}&\:\frac{{\nu\:}_{12}{E}_{2}}{1-{\nu\:}_{12}{\nu\:}_{21}}&\:0\\\:\frac{{\nu\:}_{21}{E}_{1}}{1-{\nu\:}_{12}{\nu\:}_{21}}&\:\frac{{E}_{2}}{1-{\nu\:}_{12}{\nu\:}_{21}}&\:0\\\:0&\:0&\:{G}_{12}\end{array}\right]$$	$$\:{C}_{VUMAT}=\left[\begin{array}{cc}\begin{array}{ccc}{C}_{11}&\:{C}_{21}&\:{C}_{31}\\\:{C}_{12}&\:{C}_{22}&\:{C}_{32}\\\:{C}_{13}&\:{C}_{23}&\:{C}_{33}\end{array}&\:\begin{array}{ccc}0&\:0&\:0\\\:0&\:0&\:0\\\:0&\:0&\:0\end{array}\\\:\begin{array}{ccc}0&\:0&\:0\\\:0&\:0&\:0\\\:0&\:0&\:0\end{array}&\:\begin{array}{ccc}{C}_{44}&\:0&\:0\\\:0&\:{C}_{55}&\:0\\\:0&\:0&\:{C}_{66}\end{array}\end{array}\right]$$ $\:{C}_{11},\:{C}_{22},\:{C}_{33}$​​: Stiffness in principal directions (warp, weft, transverse). $\:{C}_{12},\:{C}_{13},\:{C}_{23}$​: Poisson coupling terms. $\:{C}_{44},\:{C}_{55},\:{C}_{66}$​: Shear stiffnesses (in-plane and out-of-plane).
Shear damage	Not included natively	Explicitly programmable (​).
Stress strain Relation for orthotropic material	$$\left\{ {\begin{array}{*{20}c} {\sigma _{{11}} } \\ {\sigma _{{22}} } \\ {\sigma _{{12}} } \\ \end{array} } \right. = C_{{VFABRIC}} \left\{ {\begin{array}{*{20}c} {_{{11}} } \\ {_{{22}} } \\ {\gamma _{{12}} } \\ \end{array} } \right.$$	$$\:\left(\begin{array}{c}{\epsilon\:}_{11}\\\:{\epsilon\:}_{22}\\\:{\epsilon\:}_{12}^{el}\end{array}\right)=\left(\begin{array}{ccc}\frac{1}{(1-{d}_{1}){E}_{1}}&\:\frac{-{\nu\:}_{12}}{{E}_{1}}&\:0\\\:\frac{-{\nu\:}_{21}}{{E}_{2}}&\:\frac{1}{(1-{d}_{2}){E}_{2}}&\:0\\\:0&\:0&\:\frac{1}{(1-{d}_{12})2{G}_{12}}\end{array}\right)\left(\begin{array}{c}{\sigma\:}_{11}\\\:{\sigma\:}_{22}\\\:{\sigma\:}_{12}\end{array}\right)$$ $\:{G}_{12}:$ Shear modulus $\:{d}_{1}$: Damage in the warp direction $\:{d}_{2}$: Damage in weft direction $\:{d}_{12}:\:$ Shear damage

The VUMAT subroutine was developed in the Fortran programming language using the Intel Fortran Compiler (included in the Intel oneAPI Base Toolkit v2021.2.0^30^). Compilation was performed within Visual Studio 2019^31^and linked to Abaqus via the Intel oneAPI HPC Toolkit^32^.

**Fig. 15:**
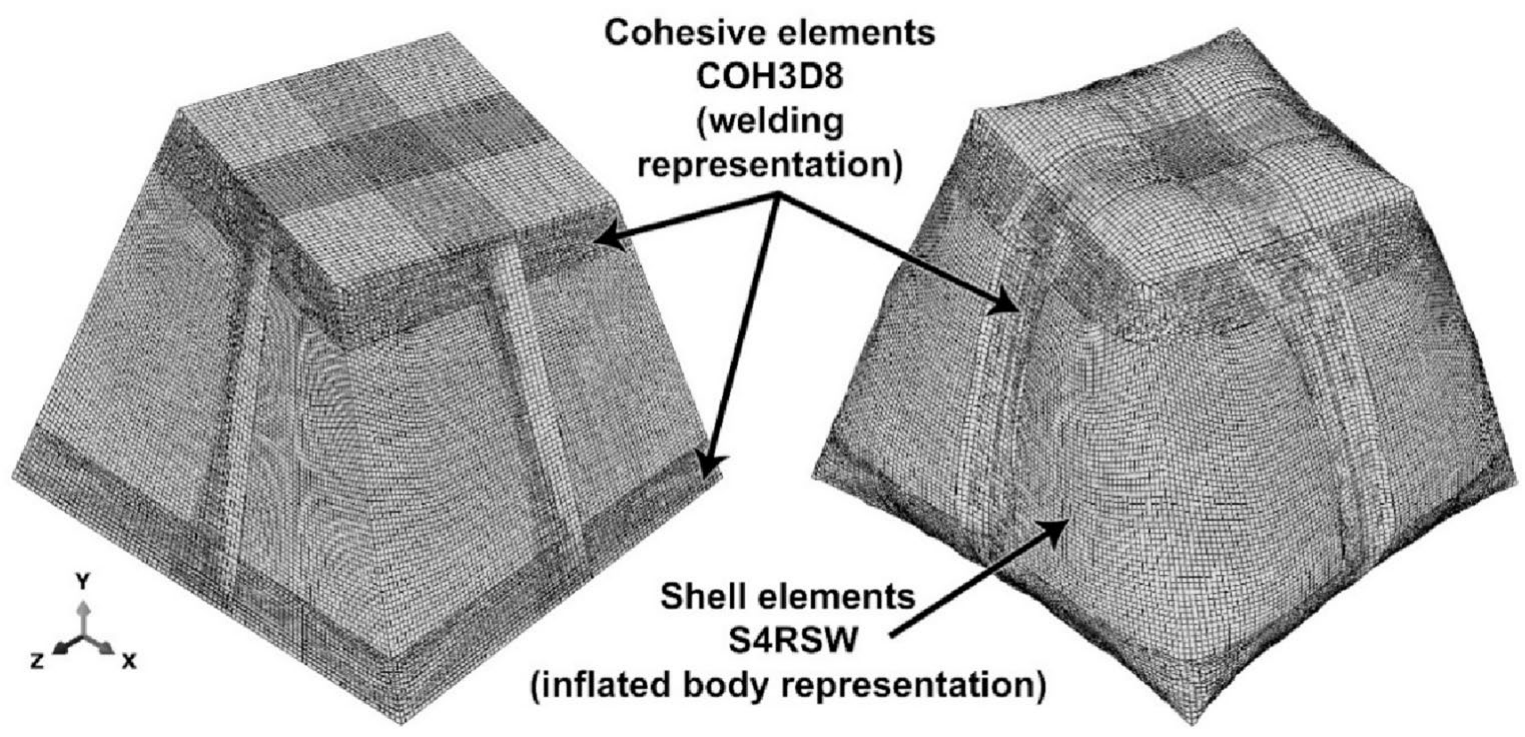
Meshed configuration and element types (left: uninflated geometry, right: inflated geometry).

S4RSW elements (warping-capable shells) were selected to ensure convergence (mesh independence verified with ± 2%)^33^, with a mesh size range between 2 and 5 mm (Fig. 15). The choice between membrane elements and shell elements (especially those allowing warping/bending) is due to their ability to simulate bending and large deformations.

#### 2.5.2. Welding representation

Surface-to-surface tie constraints are used to model weld seams between independent meshes. To capture the progressive failure of welds under peeling and shearing loads, the cohesive zone modeling (CZM) technique is employed. The cohesive meshing element type COH3D8 with 8 nodes and 4 integration points is applied to a 3D cohesive layer between the two PVC fabric layers. The variables $\:E/Knn$, $\:G1/Kss$ and $\:G2/ktt$ are obtained from the peel tension test mentioned previously. A linear traction–separation law with damage initiation (maximum stress) and energy-based evolution is used. The layer of adhesive is considered to have negligible thickness; therefore, a traction–separation response is used to define the section properties of adhesive elements^34^.

#### 2.5.3. Loading and boundary conditions

Two approaches were employed to validate the external loading behavior of the FEA model. The first involved comparing the results of quasi-static compression tests conducted on the fabricated test cases with corresponding simulations performed using the proposed model, where an internal pressure of 1.5 bar was maintained constant in both the experimental and numerical tests. The second approach focused on validating the model against experimental data from manufactured inflatable beams (cantilever supported), each 3 m in length and with diameters of 200, 250, and 300 mm, respectively. During these tests, three discrete pressure levels (0.5, 1.0 and 1.5 bar) were used to assess the mechanical behavior.

## 3. Results

### 3.1. Geometrical accuracy validation

In this section, the accuracy of the developed framework in predicting the inflated structure’s geometry was evaluated. The numerical model predicted the final shape based on the initial uninflated geometry and the applied inflation pressure. Inflated samples were suspended freely and photographed from all angles using over 200 high-resolution images captured with a Sony A7R-III DSLR camera. The images were processed in Reality Capture 1.3 software to generate dense point clouds of the inflated surfaces. The resulting 3D geometries were then compared via GOM (ZEISS) geometrical inspection software.

#### 3.1.1. [2D] symmetric pillow

**Fig. 16:**
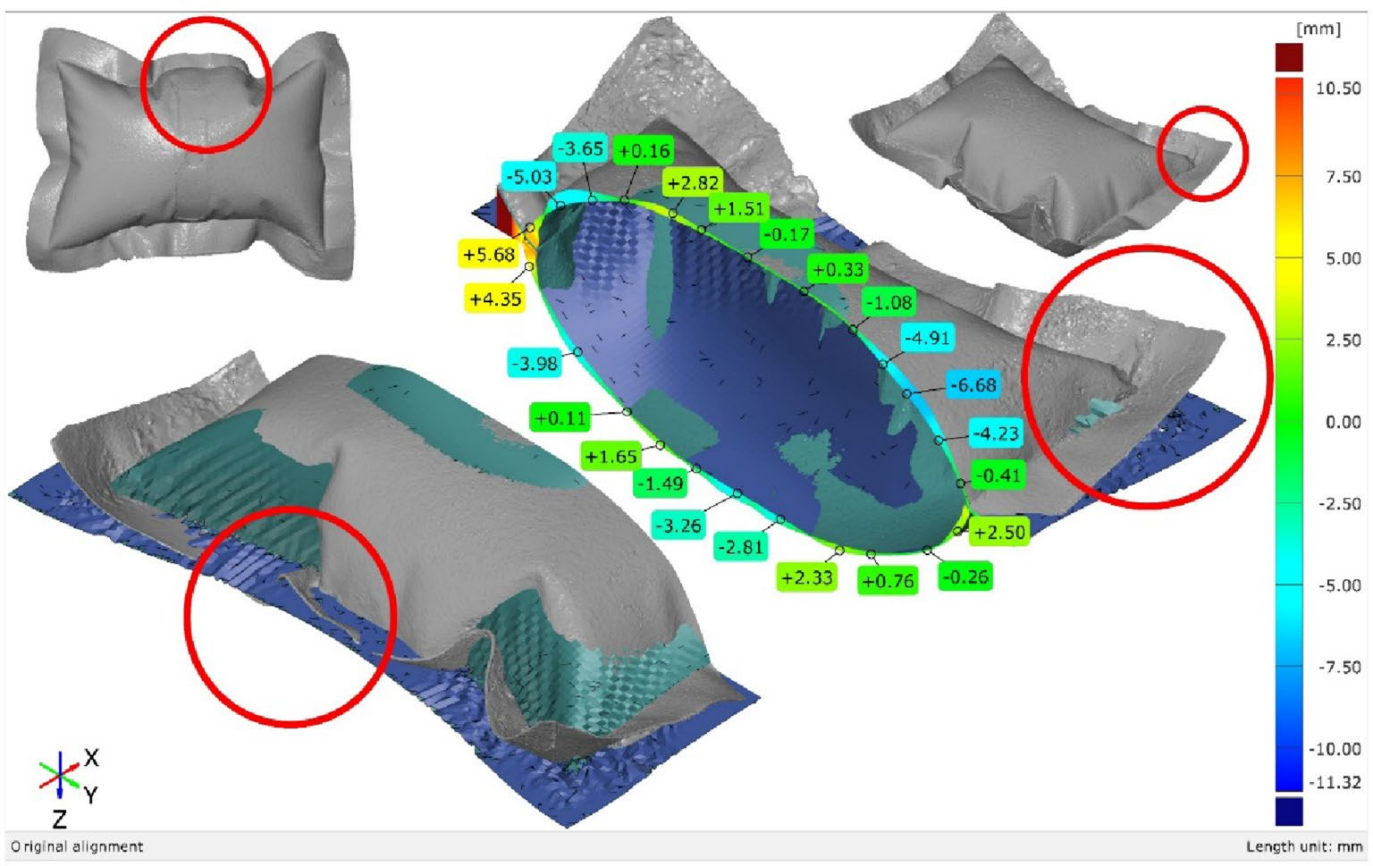
Transverse cross-sectional geometrical error of the 3D scanned inflatable 2D Pillow (1.5 bar) pressure compared with the model predicted via [GOM Inspect] software.

The comparison between the 3D-scanned geometry (Supplementary Figs. 1–2) and the numerical predictions shows deviations between − 13.79 and + 1.31 mm for the lateral cross-section (Supplementary Fig. 3) and − 7.2 to + 5.68 mm (Supplementary Fig. 4) in the transverse cross-section as shown in (Fig. 16). The larger lateral discrepancy is attributed to welding defects or poor T-peel resistance, these irregularities highlight the influence of manufacturing quality on the inflated shape and motivate further exploration of more complex 3D geometries. A limitation of the current FEA model is its inability to accurately predict behavior at corner edges, which led to the rejection of the outer-edge welding approach (peel adhesion) initially considered for other samples.

Further details of the finite element mesh, photogrammetry procedure, detailed geometric comparisons, and the influence of welding defects – highlighted in the annotated regions (Fig. 16) – are presented in Supplementary Note 1.

#### 3.1.2. [3D] symmetric cuboid with inner stiffener

**Fig. 17:**
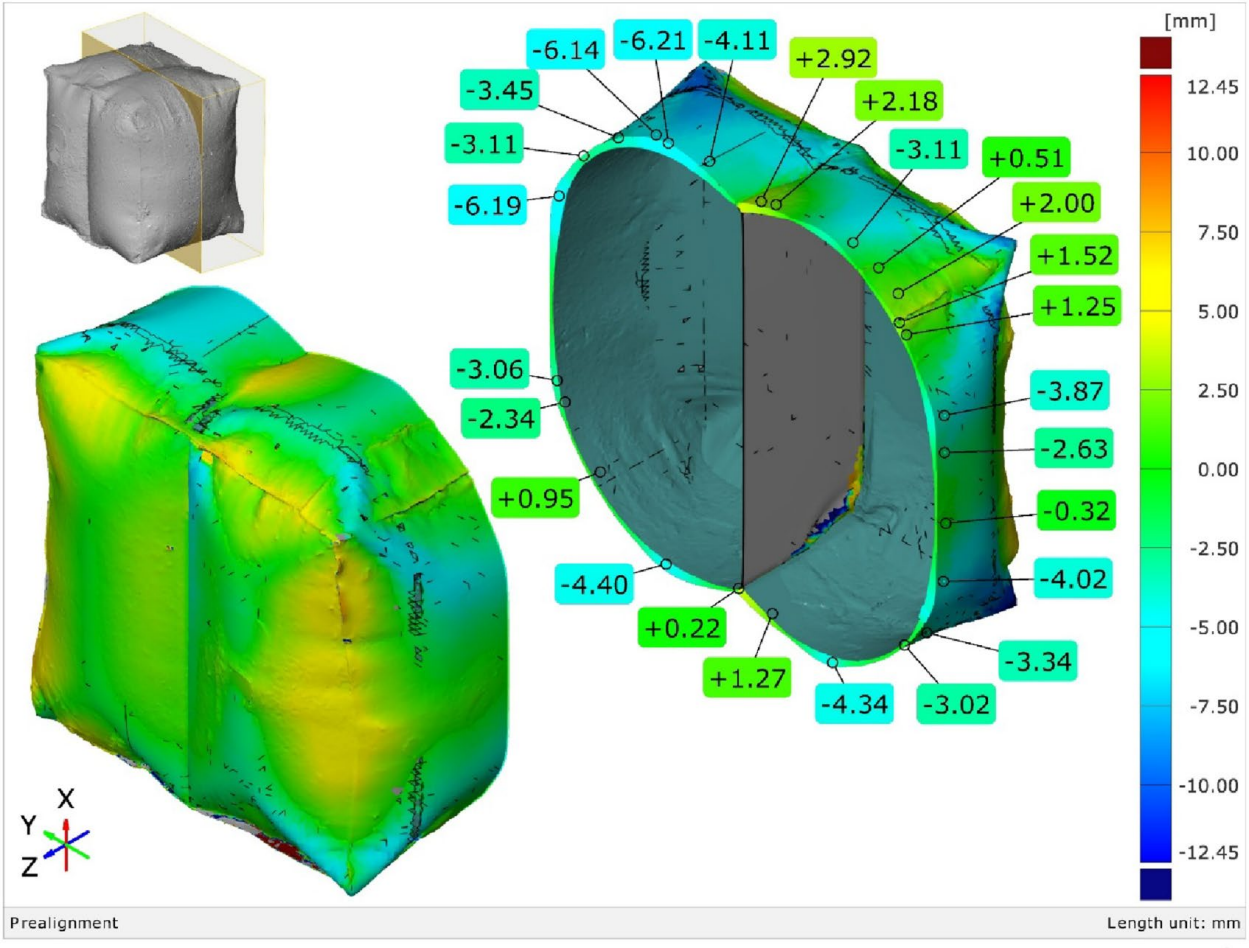
Geometrical error of the 3D scanned inflatable cuboid at (1.5 bar) inflation pressure, compared with the model predicted model via [GOM Inspect] software.

The FEA model was used to validate the shape prediction accuracy of the inflated symmetric cuboid. The numerical model consisted of 181,655 elements (see Supplementary Note 2). Comparison between the predicted and 3D-scanned geometries showed maximum deviations of −6.21 mm and + 2.94 mm across the transverse section (Supplementary Figs. 5–6), indicating very good agreement between simulation and experiment as illustrated in (Fig. 17).

#### 3.1.3. [3D] asymmetric lofted twisted structure

**Fig. 18:**
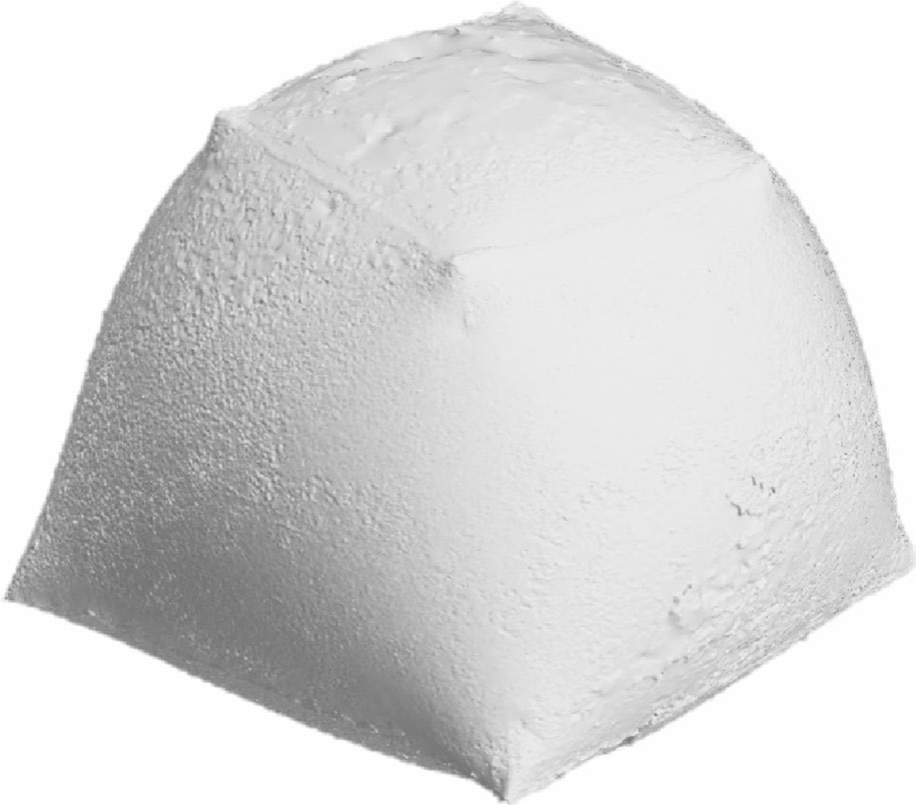
3D Scanned inflated Model of the twisted-lofted geometry without any inner stiffeners.

The FEA model for this case comprises 230,470 elements. The 3D scanning photogrammetry process utilized 382 photographs, resulting in a point cloud of 1,749,218 points. The initial mesh reconstruction generated 17 million faces, which were subsequently optimized to 1.28 million polygons (Fig. 18).

**Fig. 19:**
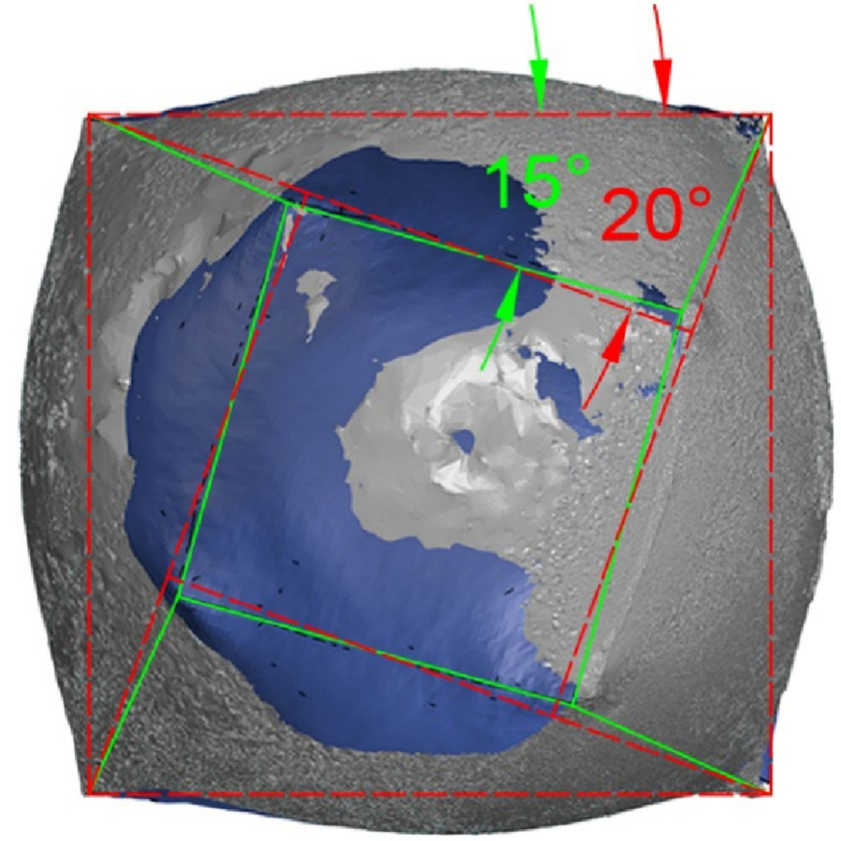
Effect of the inflation process on the twist angle of the geometry (no stiffeners shape).

Notably, the twist angle between the base and the tip decreased from 20° to 15° (Fig. 19). This relaxation behavior is influenced by internal pressure, suggesting the need for further investigation across a wider range of pressure values to better characterize this relationship.

**Fig. 20:**
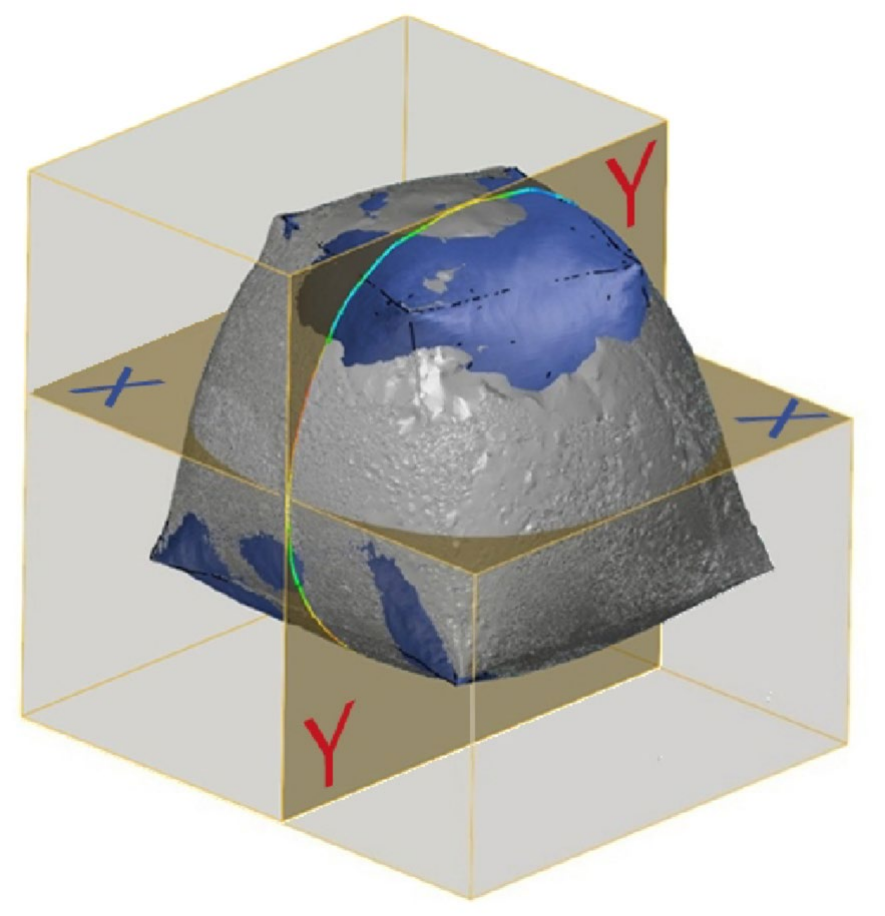
Inspection sections (X and Y planes).

**Fig. 21:**
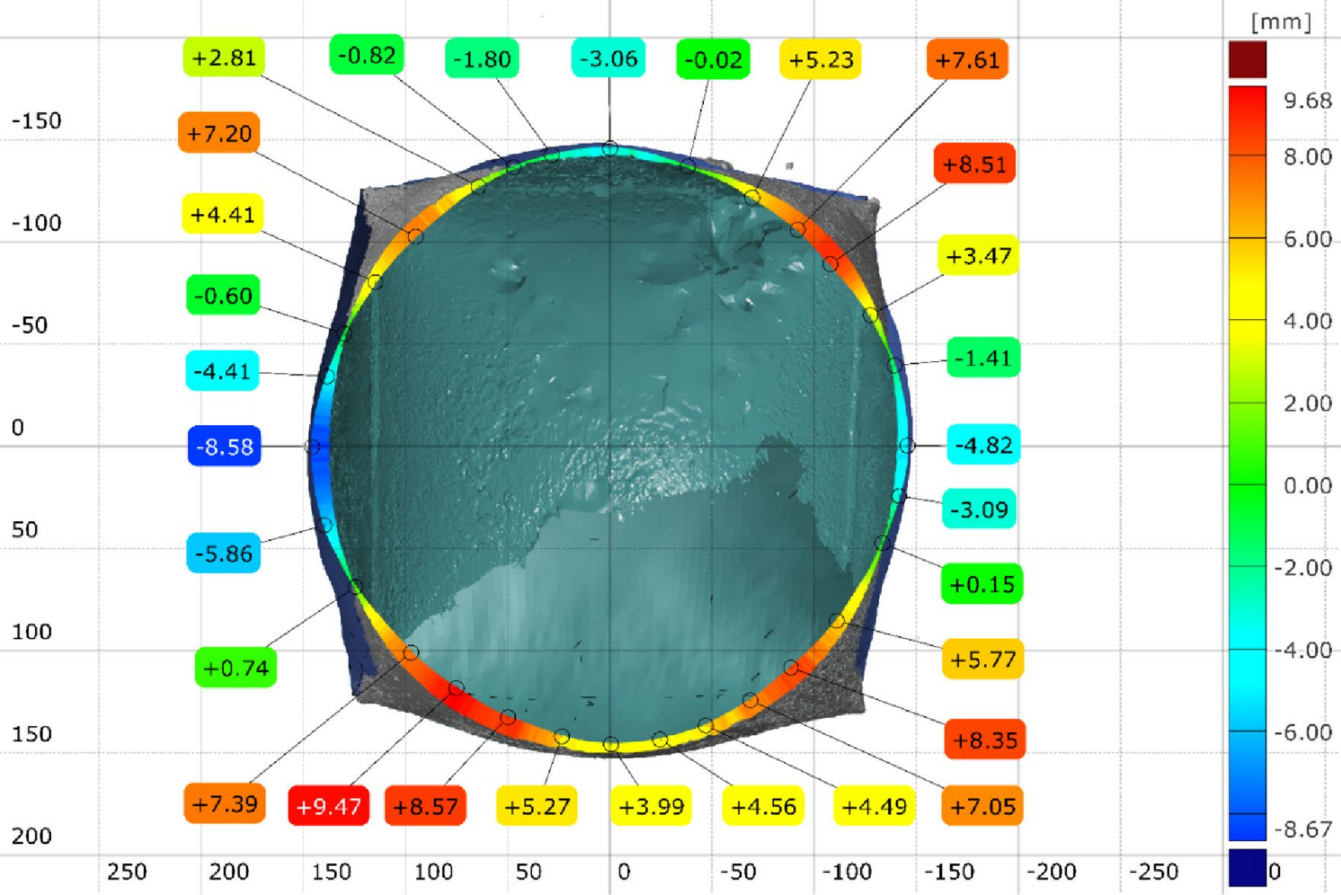
Geometrical errors on the X-plane section of the inflated twisted shape without inner stiffeners.

**Fig. 22:**
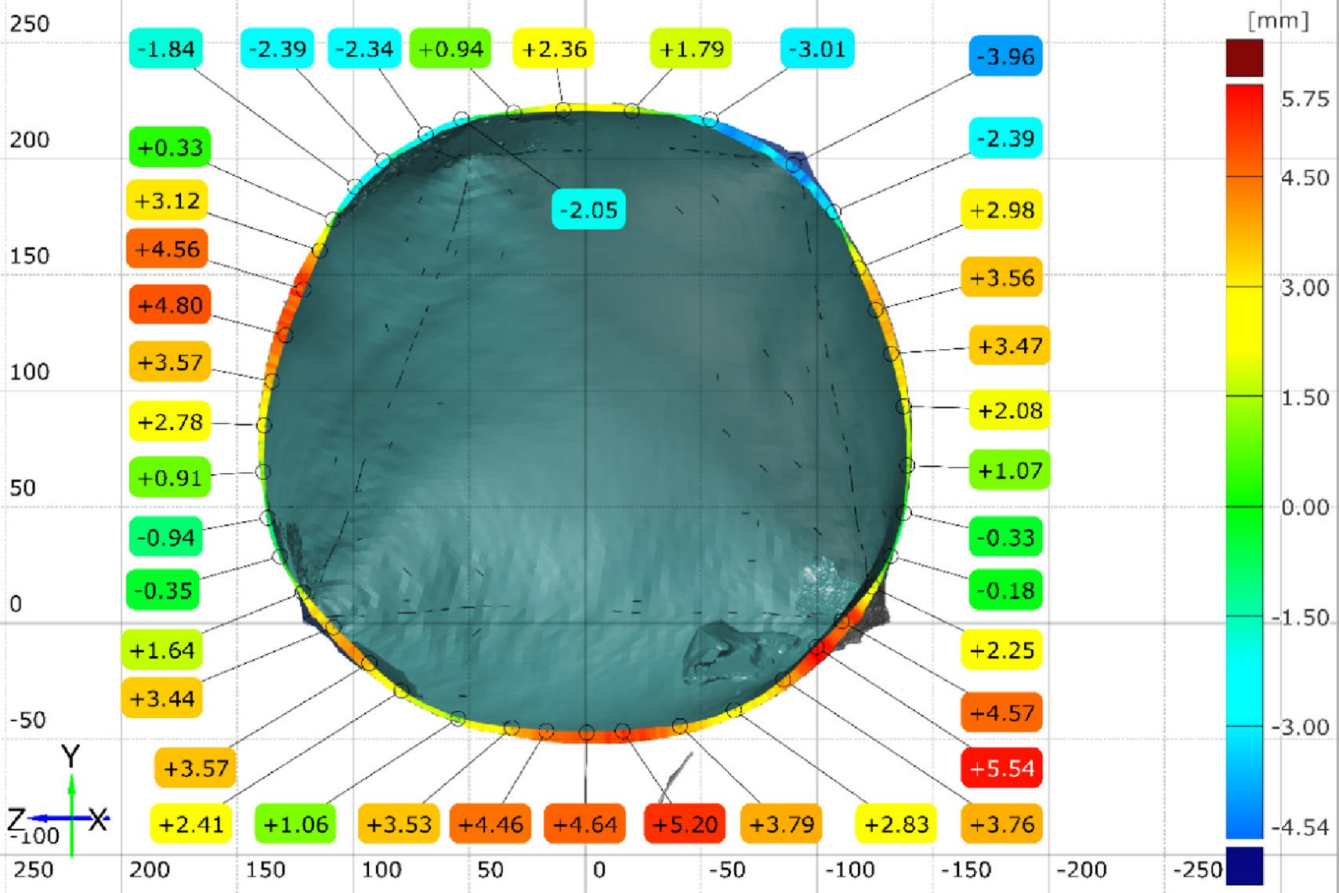
Geometrical errors on the Y-plane section of the inflated twisted shape without inner stiffeners.

The inspection sections are chosen to be at the x-x plane 50 mm offset from the base of the initial uninflated geometry. And the Y-Y plane is aligned with the axis of symmetry of the shape (Fig. 20), and the deviation error is plotted at (Fig. 21) and (Fig. 22).

#### 3.1.4. [3D] asymmetric lofted twisted structure with inners stiffeners

**Fig. 23:**
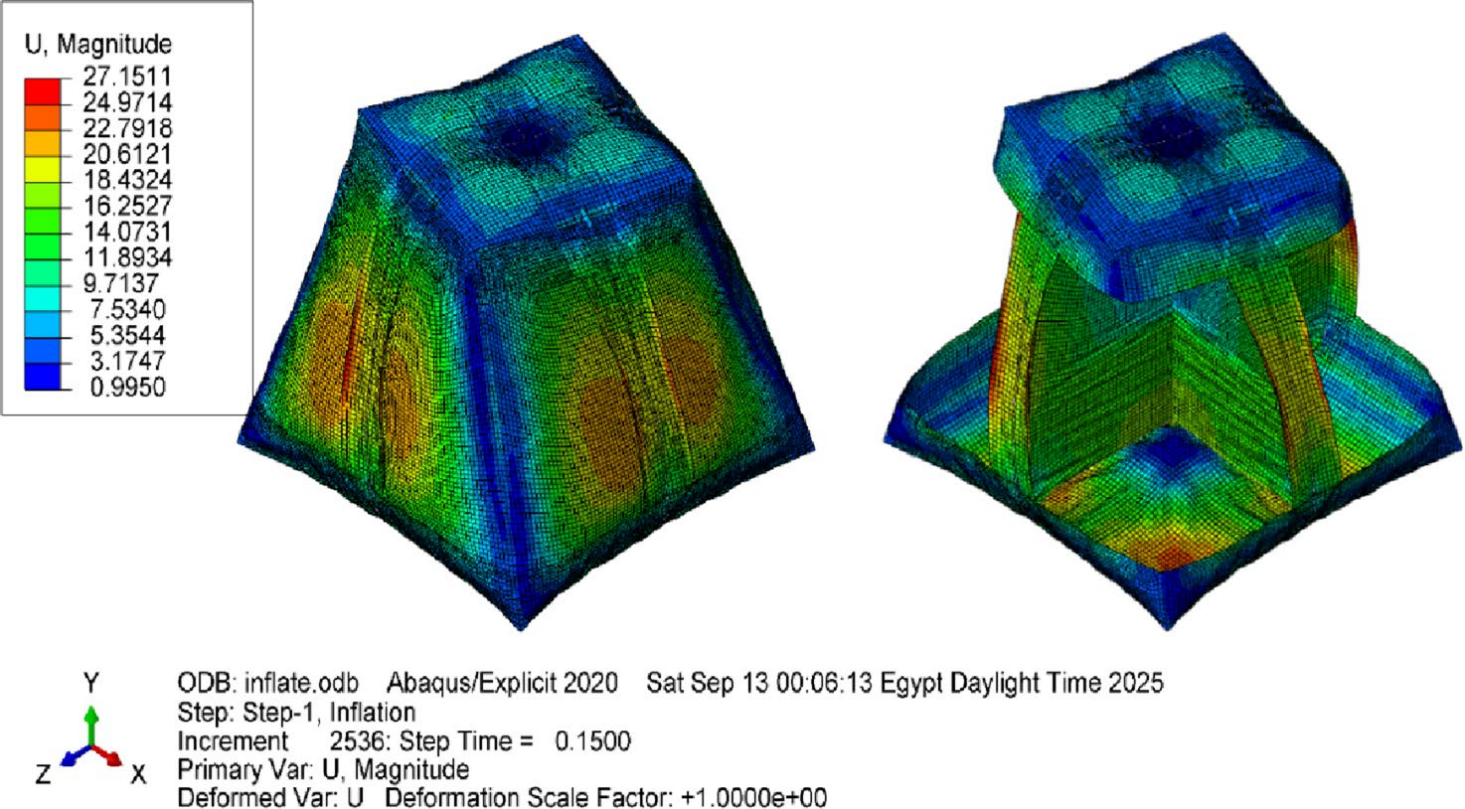
Deformations of the twisted and lofted shape, in addition to the right configuration of the inner stiffeners. Units are in mm.

**Fig. 24:**
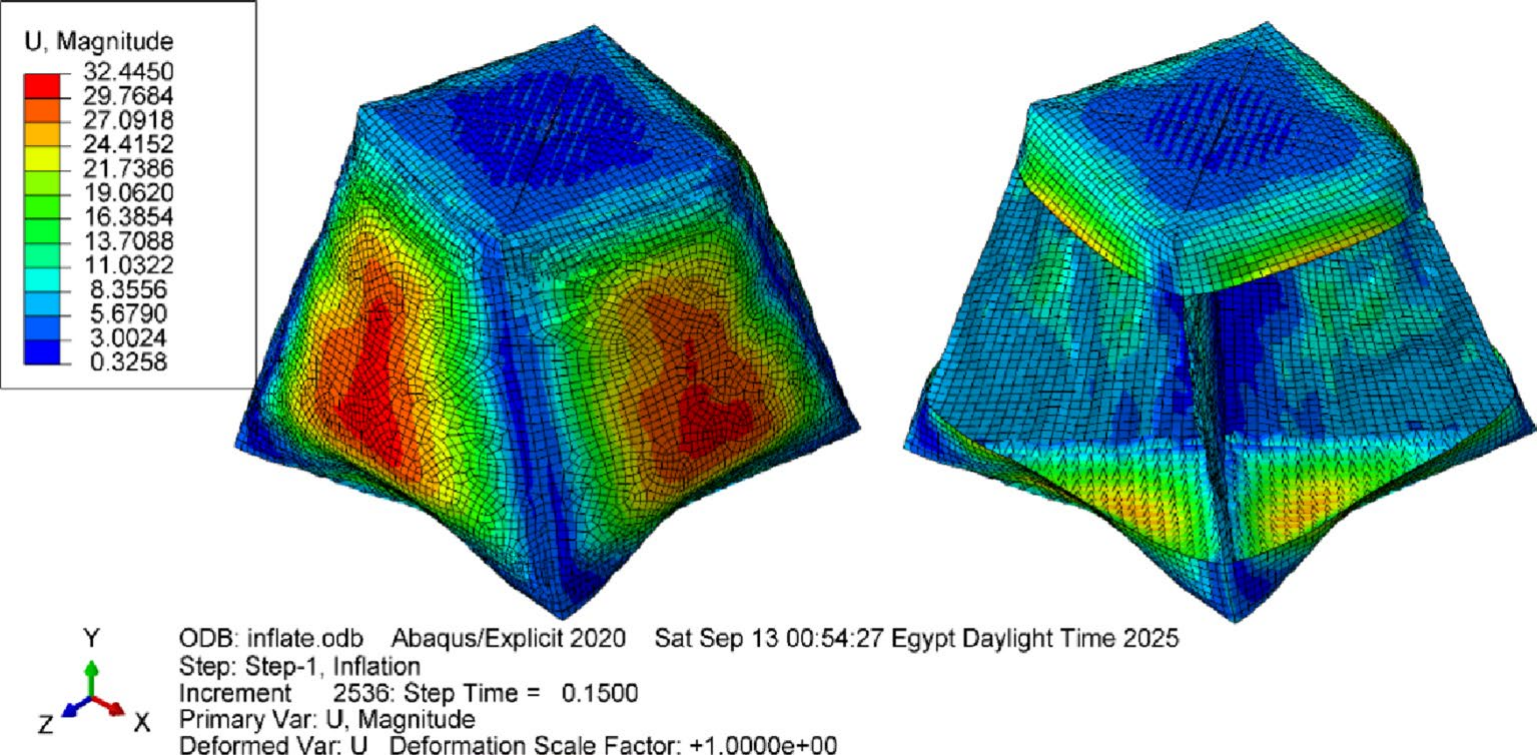
Deformations of the twisted and lofted shape in addition to the poor configuration of the inner stiffeners (diagonal arrangement), Units are in mm.

**Fig. 25:**
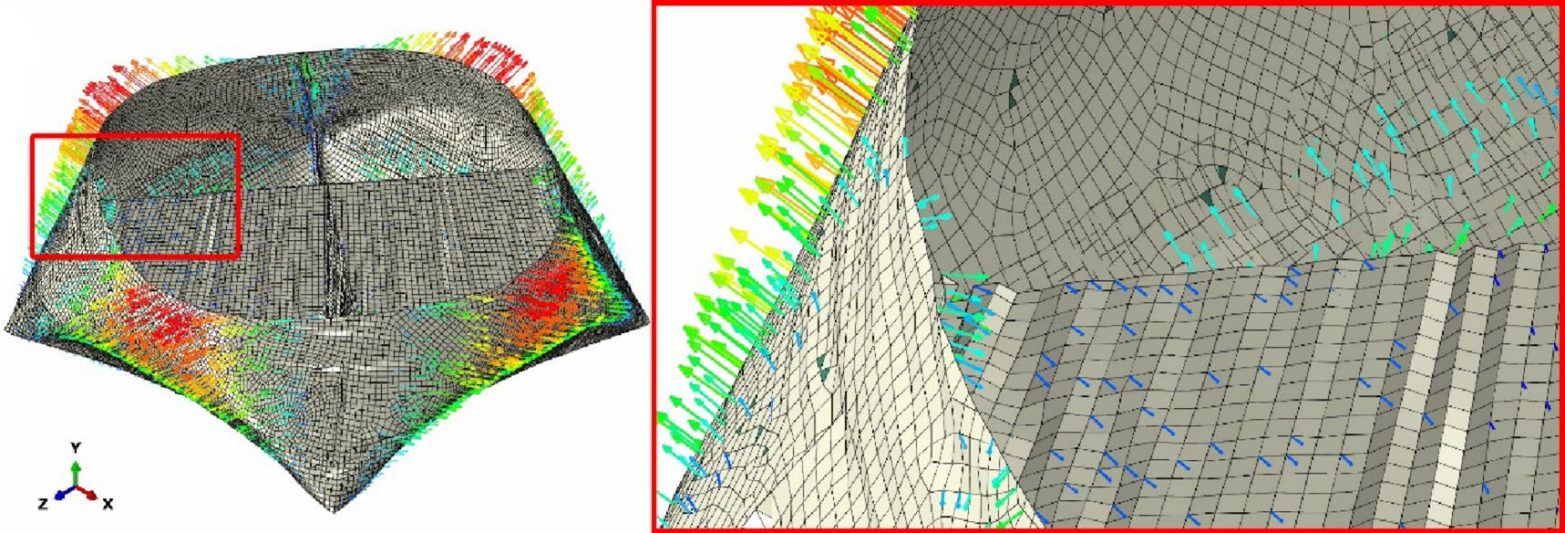
The wrinkling of the diagonal stiffeners and the corresponding load directions acting on each element.

In this model, the inner stiffeners configuration was selected using the predicted shape (Fig. 23), considering the limitations of a diagonal stiffener arrangement, where those two stiffeners are welded diagonally to the geometry and their objective is to reduce the maximum deformation of the inflated shape at the same inner pressure (Fig. 24). The configuration of the diagonal stiffeners proved to be less effective because, due to their orientation, they were primarily subjected to compressive membrane stresses. Since the PVC-coated fabric cannot sustain compression, these regions exhibited local wrinkling, causing the stiffeners to lose tension and become structurally less active. As a result, the diagonal configuration contributed minimally to the overall shape stability compared with the longitudinal and transverse arrangements. (Fig. 25) illustrates the onset of compressive wrinkling along the diagonal stiffeners and the corresponding load directions acting on each element.

The results showed that the initial twist angle 20° decreases to 16° for the given model dimensions and inflation pressure due to fabric relaxation and stress redistribution (Fig. 27). Comparing this result to the previous case without stiffness shows the effect of the stiffness distribution in the inflated structures.

**Fig. 26:**
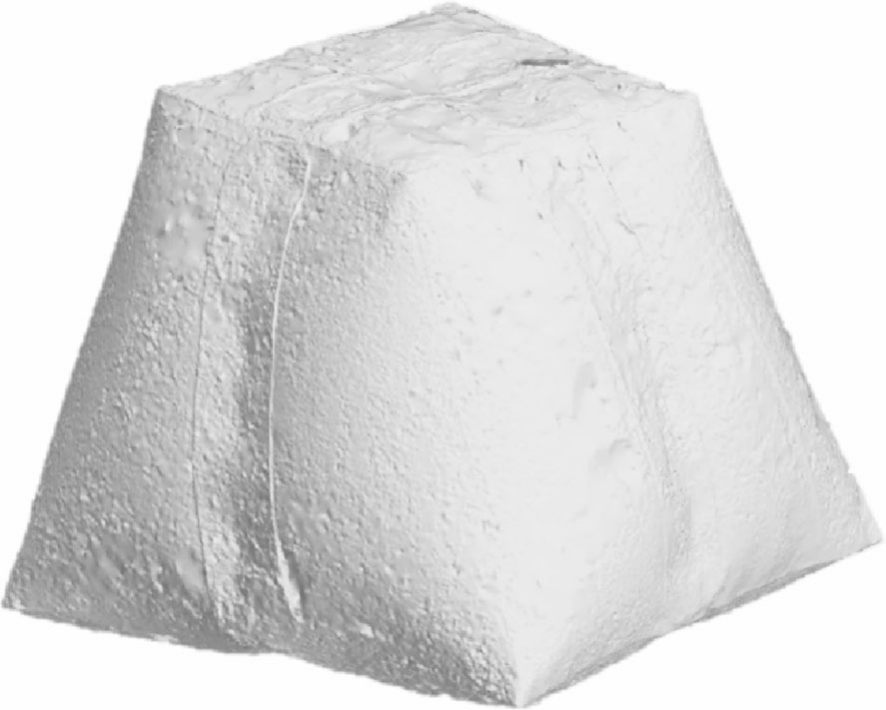
[3D] Scanned inflated Model of the twisted-lofted geometry with two inner stiffeners.

**Fig. 27:**
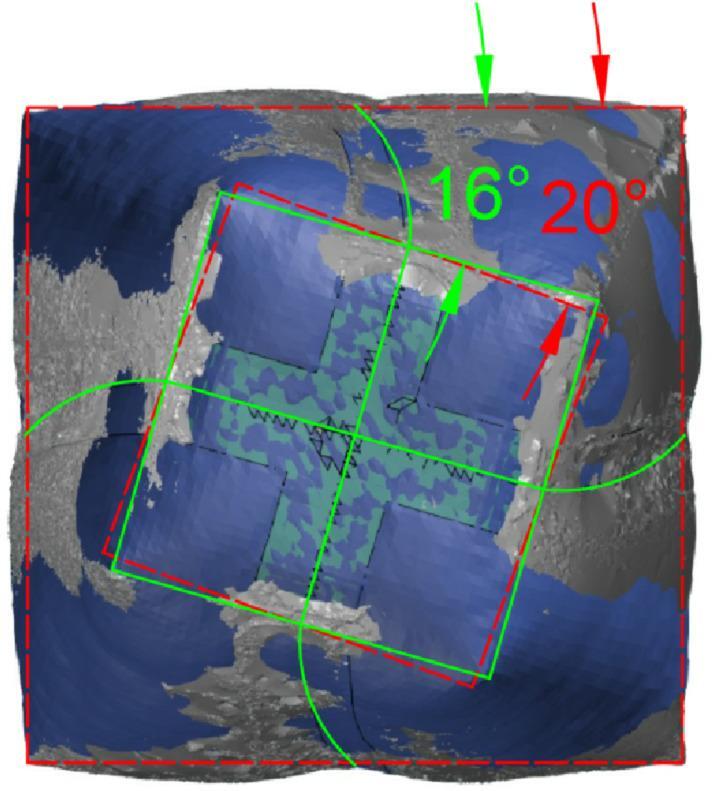
Effect of the inflation process on the twist angle of the geometry.

**Fig. 28:**
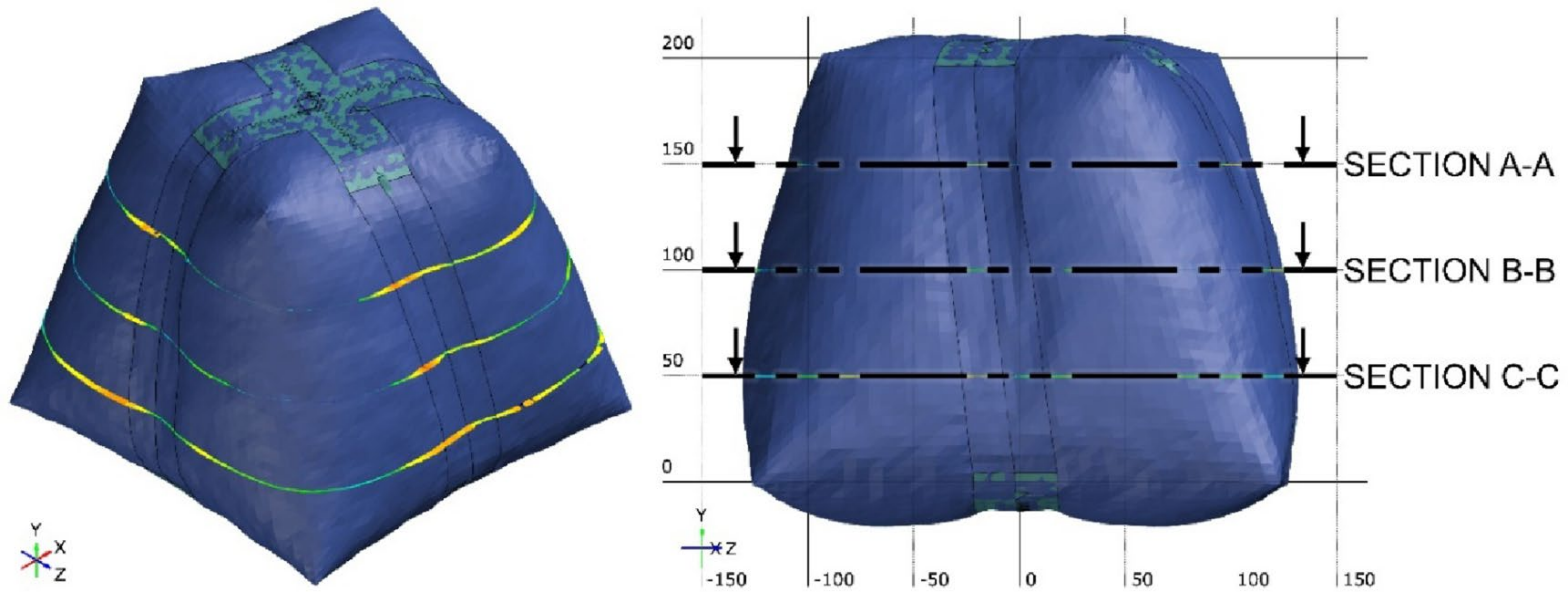
Distribution of the inspection sections.

**Fig. 29:**
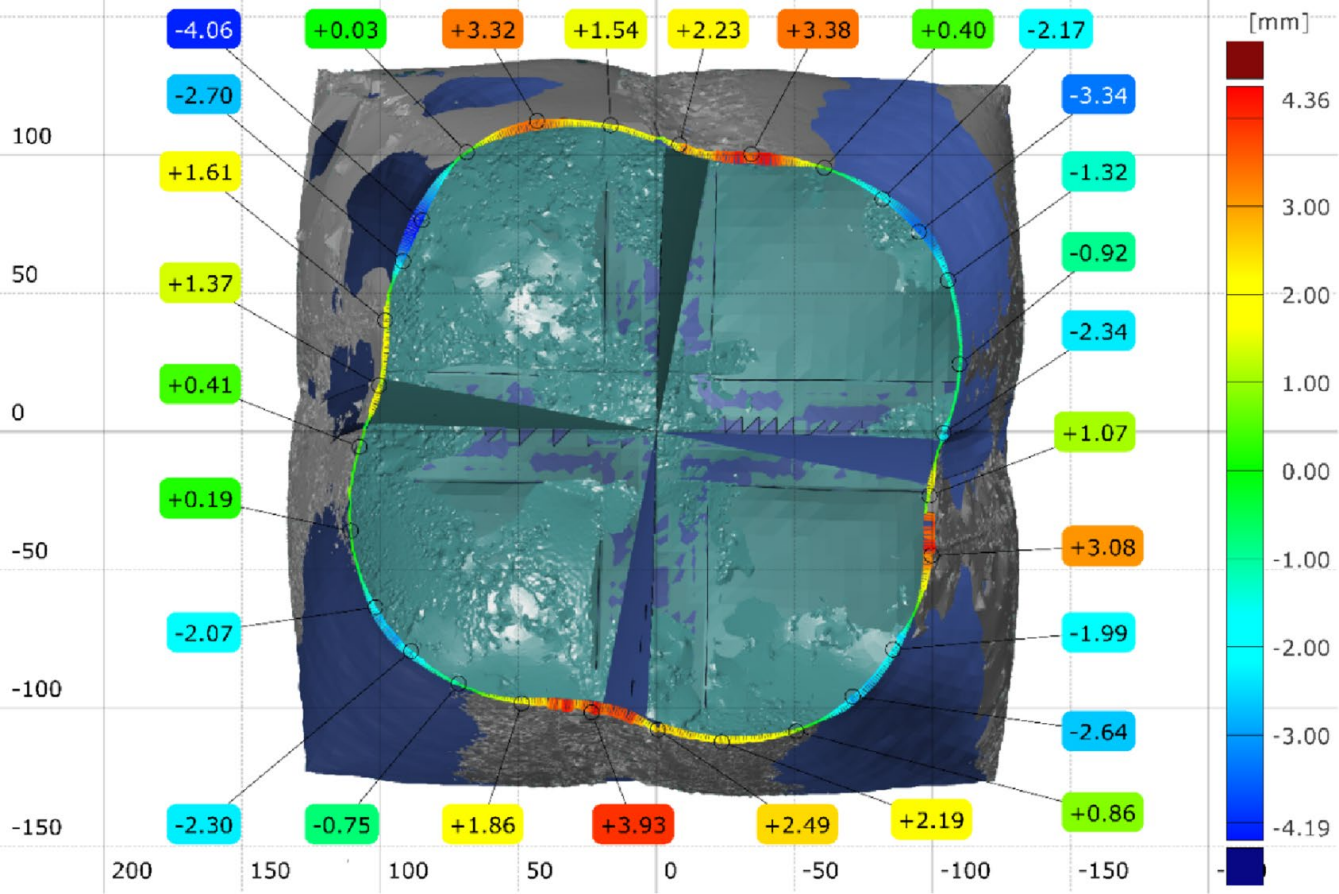
Inspection section A-A for the inflated twisted shape with inner stiffeners.

**Fig. 30:**
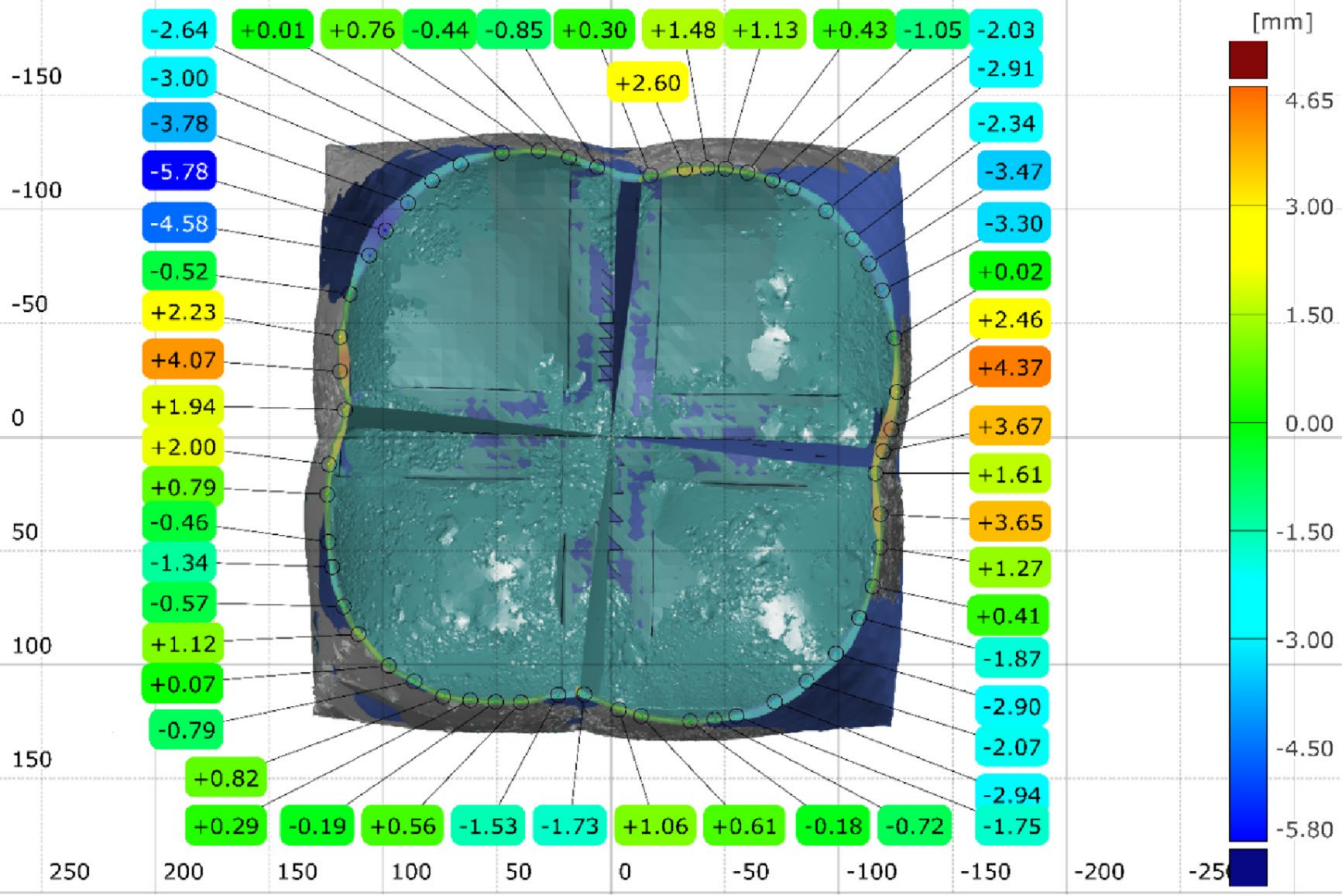
Inspection section B-B for the inflated twisted shape with inner stiffeners.

**Fig. 31:**
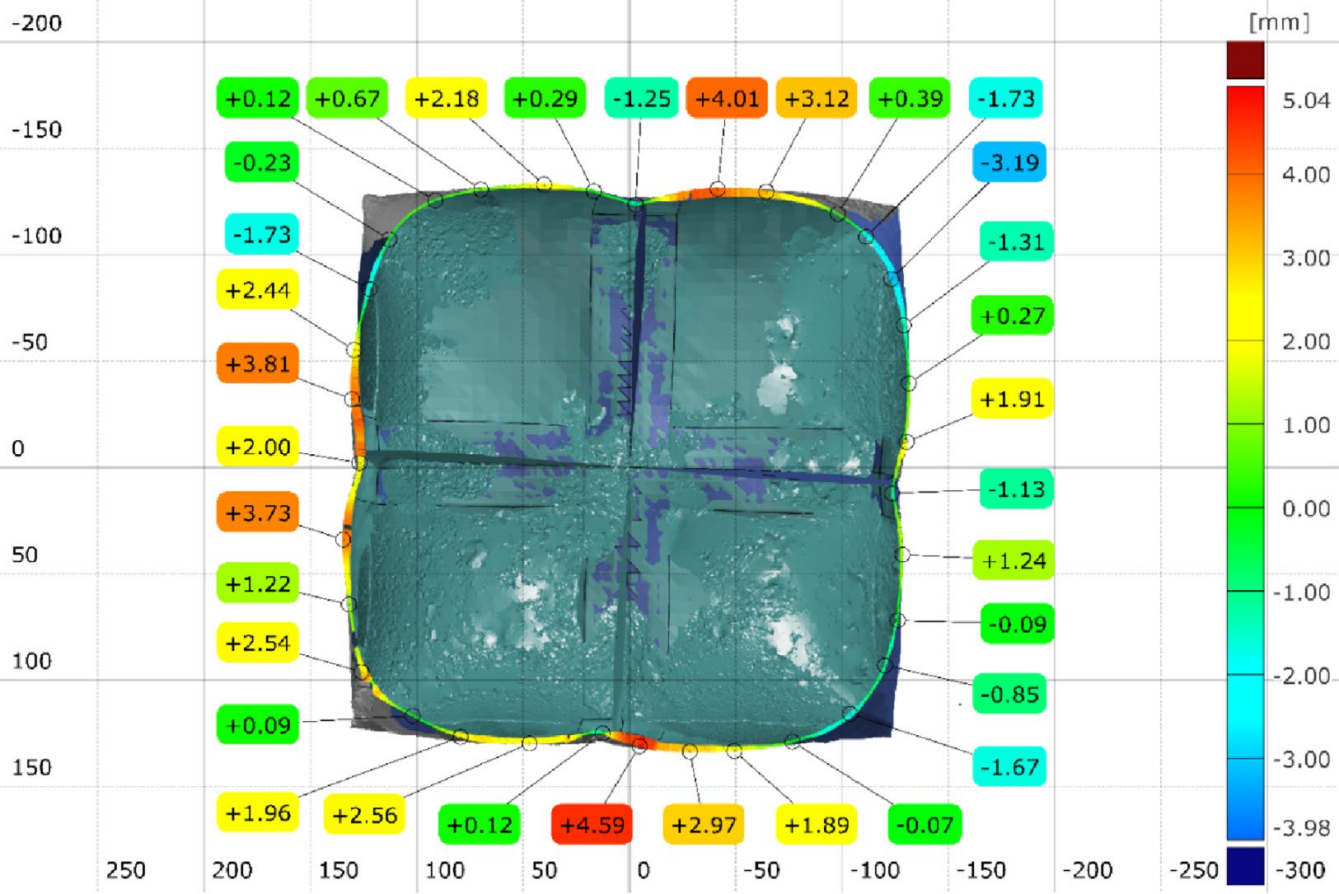
Inspection section C-C for the inflated twisted shape with inner stiffeners.

**Fig. 32:**
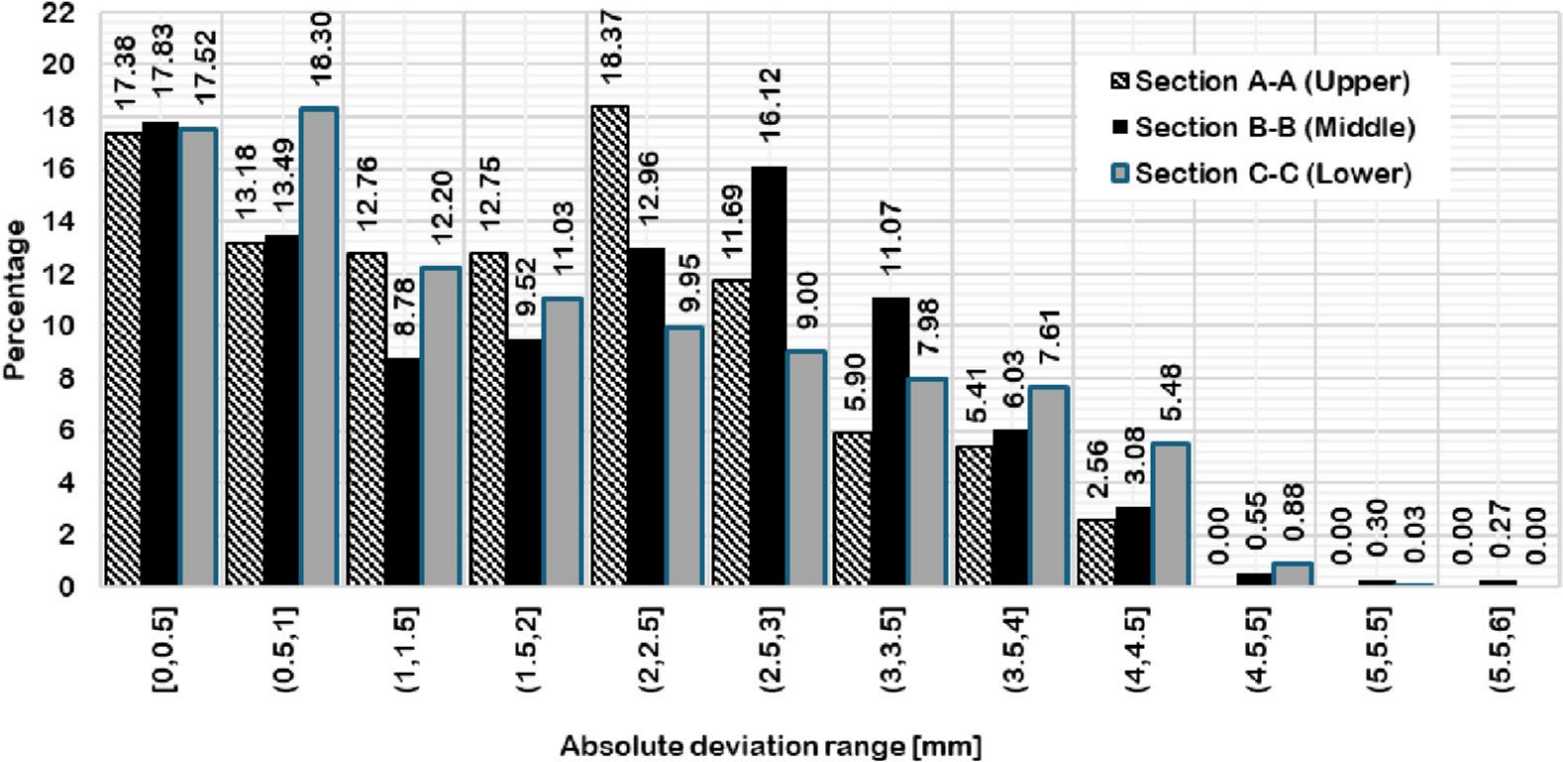
Absolute values for the deviation ranges versus their percentages for each section.

Three inspection sections are chosen to capture the deviation of the actual 3D scanned surface (Fig. 26) from the FEA generated surface. These sections are offset at equal distances of 50 mm and distributed so that the middle section B-B is consistent with the middle of the total initial height (200 mm) (Fig. 28). The deviation labels are then plotted on each section in (Fig. 29), (Fig. 30) and (Fig. 31). The ranges for the absolute values of those deviations are plotted in the histogram at (Fig. 32) with RMSE = 2.29244.

### 3.2. Mechanical performance validation

#### 3.2.1. Compression testing of inflatable free-form geometries

**Fig. 33:**
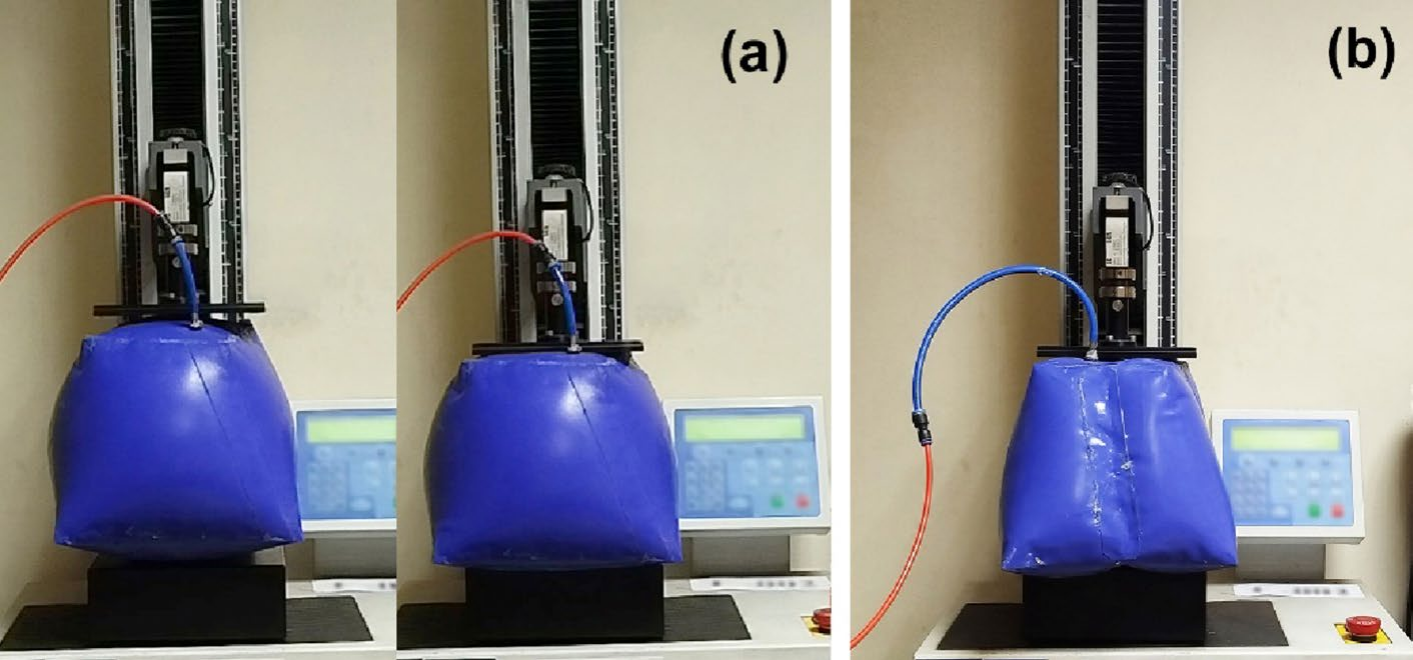
Compression test for (**a**) the inflated twisted geometry (test case 3) and (**b**) the inflated twisted geometry with internal stiffeners (test case 4).

To experimentally validate the structural performance of the inflatable free-form geometries, quasi-static compression tests were carried out on the fabricated third and fourth test cases.

(Fig. 33) under controlled internal pressure 1.5 bar using a pressure regulator. Each part was clamped at its base to replicate the boundary conditions used in the FEA model, and a vertical displacement was applied at the free end while simultaneously recording load and displacement. All compression tests were performed using a universal testing machine^35^with a capacity of 5kN, and the results were generated by the machine’s integrated data acquisition software.

**Fig. 34:**
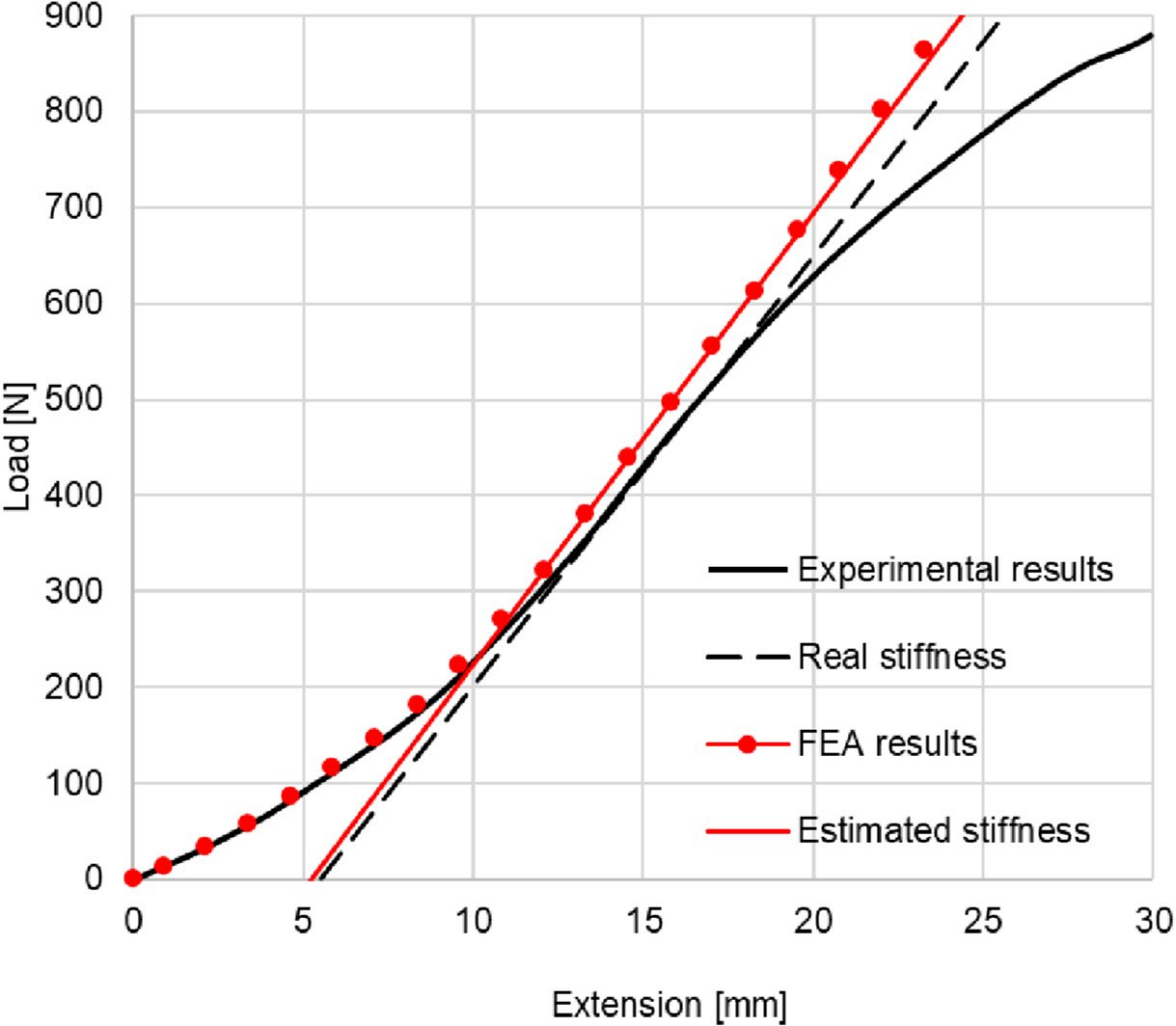
Load-elongation curve generated experimentally and using FEA simulation on test case 4.

**Fig. 35:**
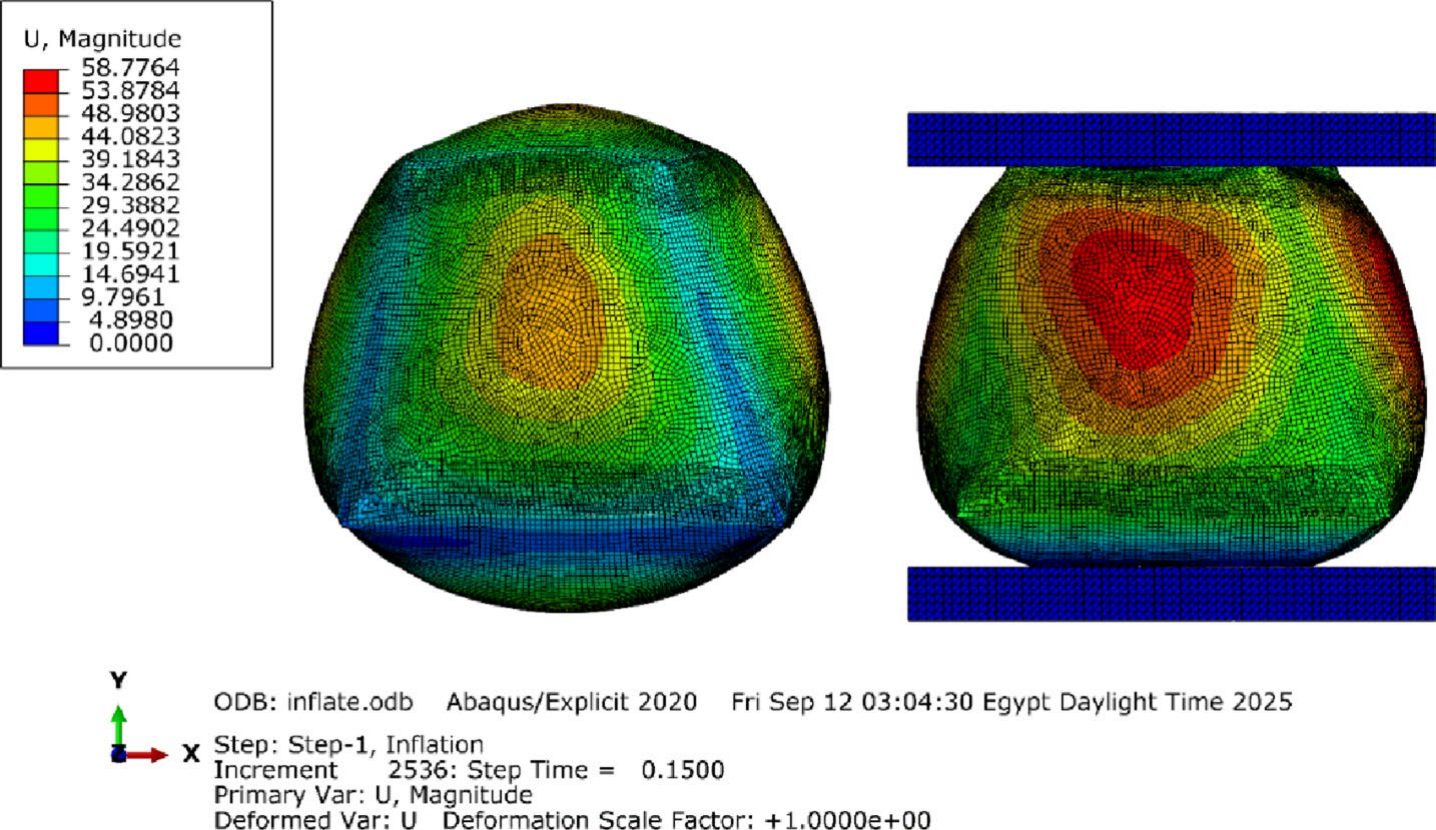
Deformations of the inflated twisted and lofted shape after undergoing compression test simulated in Abaqus. Units are in mm.

The resulting load–deflection curves (Fig. 34) provide direct measurements of stiffness and nonlinear response. Visual inspection and video recordings identified the onset of wrinkling and local buckling, correlating with strain distributions predicted by the FEA framework. Compression test results were compared with FEA simulations (Fig. 35) to estimate stiffness in both cases. These tests extend validation beyond geometry, demonstrating quantitative agreement between the model and the mechanical behavior of the complex inflatable structure.

#### 3.2.2. Simple beam validation

The proposed framework is then compared with the experimental results presented by S. Okda et al.^36^, in which inflated straight beams were tested with different geometries, internal pressures, and load conditions. Three beams with diameters of 200, 250 and 300 mm and fixed length of 3300 mm, were cantilever-mounted and subjected to lateral loads. These beams were manufactured with the same material and thickness used for geometrical validation. Three different inflation pressures are applied: 0.5, 1.0, 1.5 bar. Results from this study are represented as load–deflection curves for each beam under different conditions as shown in (Fig. 38).

**Fig. 36:**
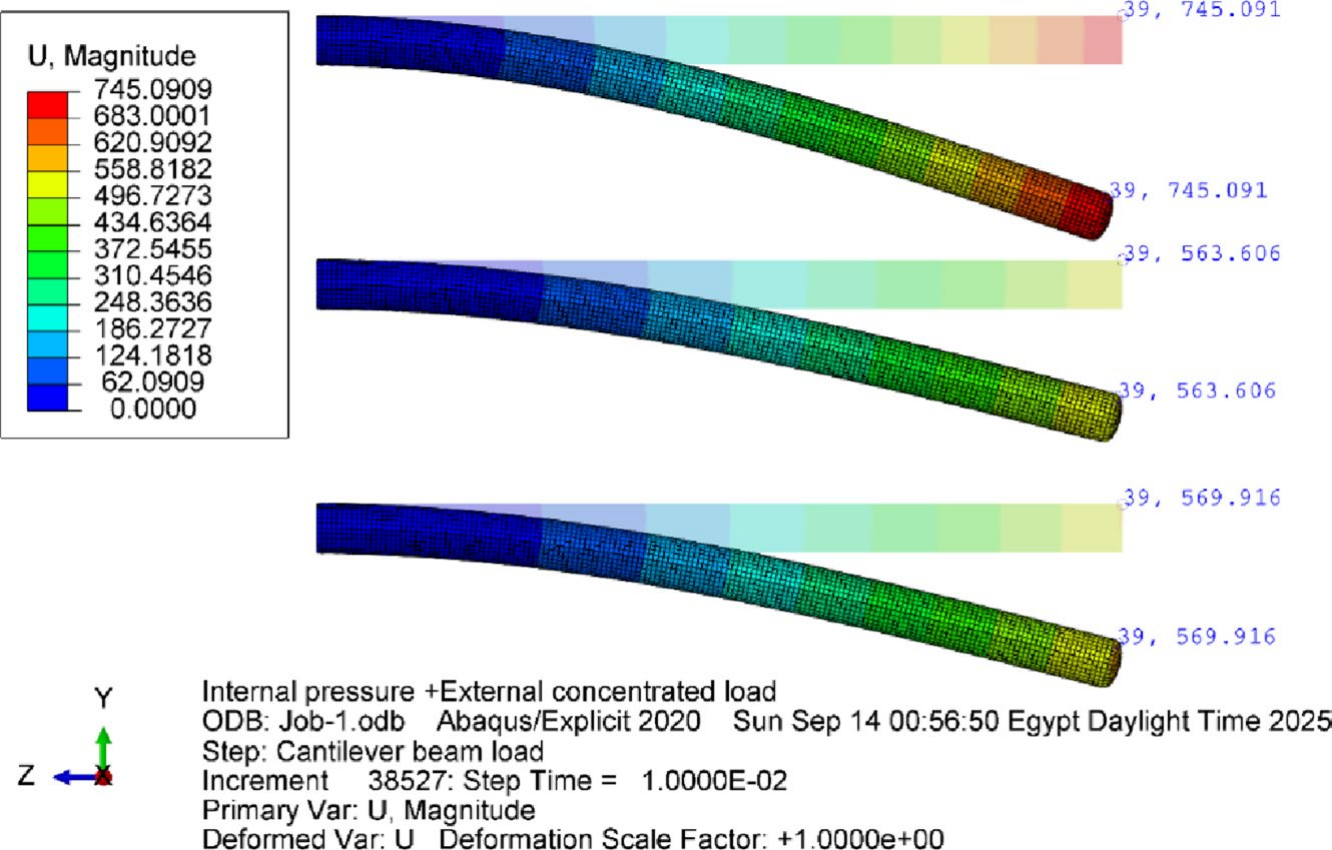
Deflections of a 200 mm diameter cantilever inflatable beam (3300 mm long) under 95 N force loading up, 65 N (middle) and 36 N (down) at pressure values of 1.5,1 and 0.5 bar respectively.

**Fig. 37:**
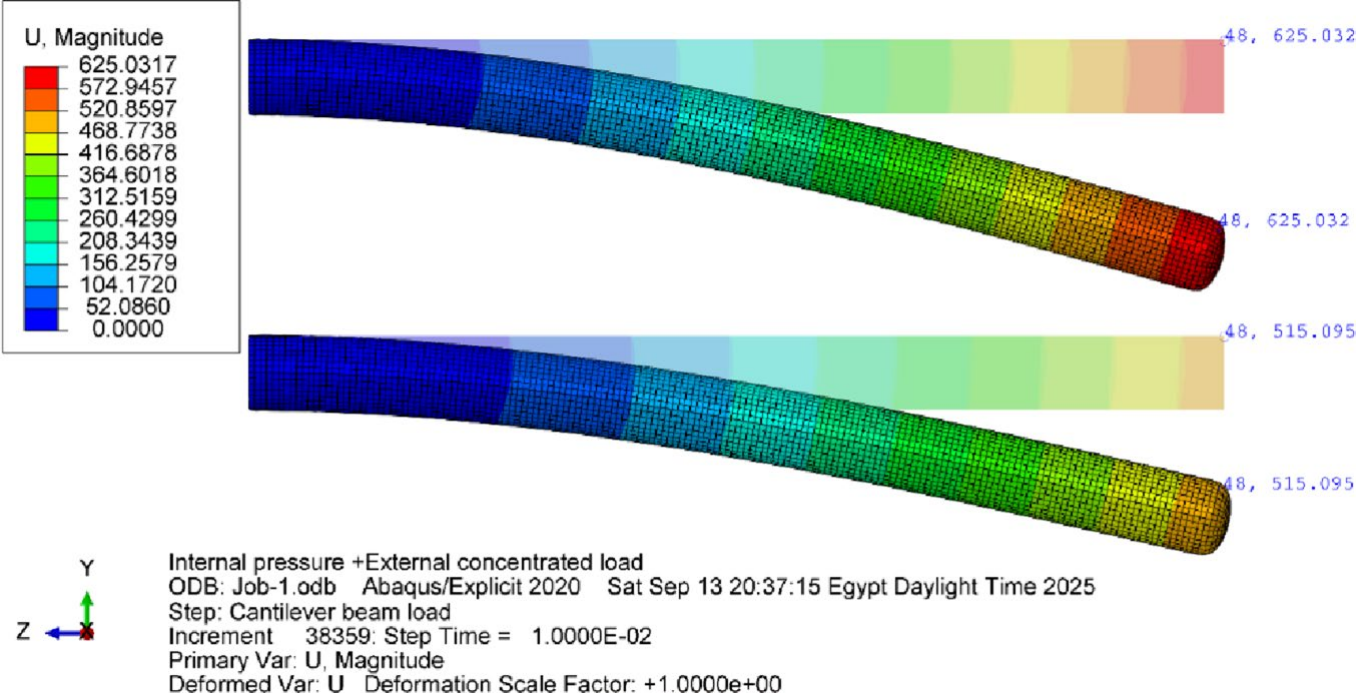
Deflections of a 250 mm diameter cantilever inflatable beam (3300 mm long) under 150.5 N force loading (up) and 90 N (down) at pressure values of 1 and 0.5 bar respectively.

The simulation model was developed to replicate these experimental conditions. (Fig. 36) and (Fig. 37) present representative results from the numerical predictions, demonstrating the model’s capability to capture the beam behavior across different configurations.

**Fig. 38:**
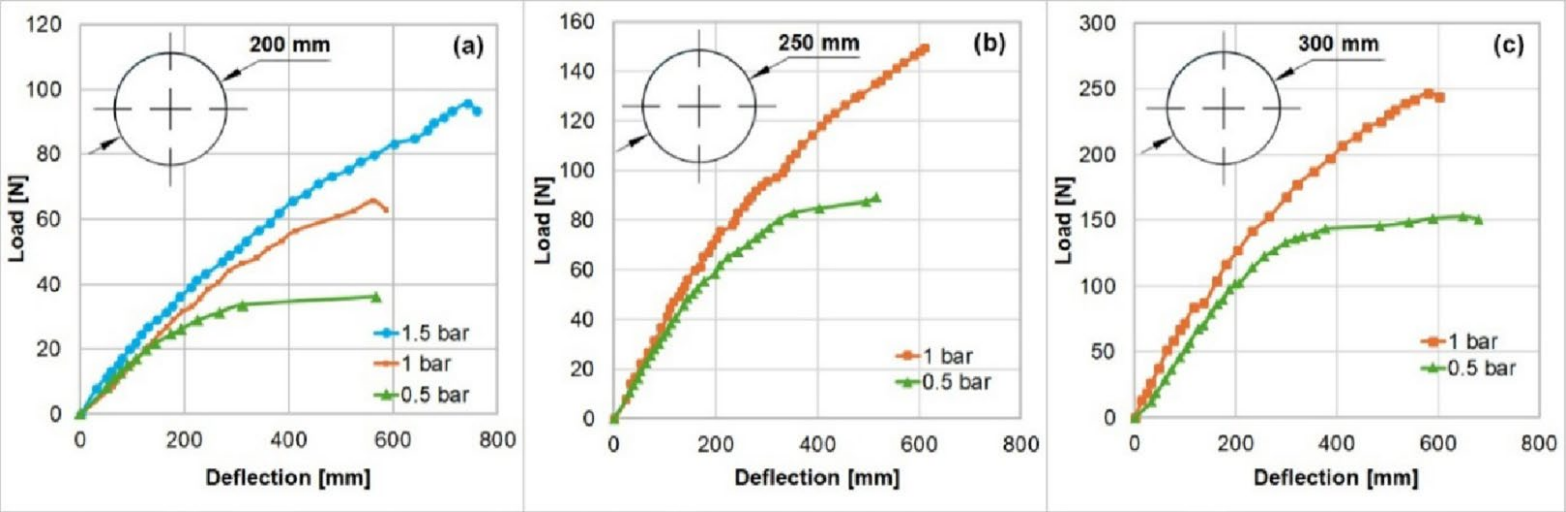
The load–deflection curves of the three inflatable beams with diameters (**a**) 200 mm, (**b**) 250 mm and (**c**) 300 mm at three inflation pressures 1.5, 1.0 and 0.5 bar.

(Table 4) summarizes the predictions of the model for the deflection before wrinkling compared with the experimental results.

**Table 4:** Stiffness and maximum load values of the three inflatable beams under different inflation pressures - Practical results compared with the simulated results.

Beam Diameter (mm)	Inflation pressure (bar)	Stiffness (*N*/m)	Maximum Load (*N*)	Deflection before Wrinkling/practical (mm)	Deflection before Wrinkling Simulated (mm)	Error %
200	0.5	140.57	36	570	571.56	+ 0.27
	1	159.74	65	563.5	563.06	− 0.08
	1.5	206.72	95	745	746.59	+ 0.21
250	0.5	347.5	90	515	539.51	+ 4.76
	1	382.6	150.5	625	623.75	−0.2
300	0.5	520	153.5	650	678.48	+ 4.38
	1	779.2	246	585	566.39	−3.18

**Fig. 39:**
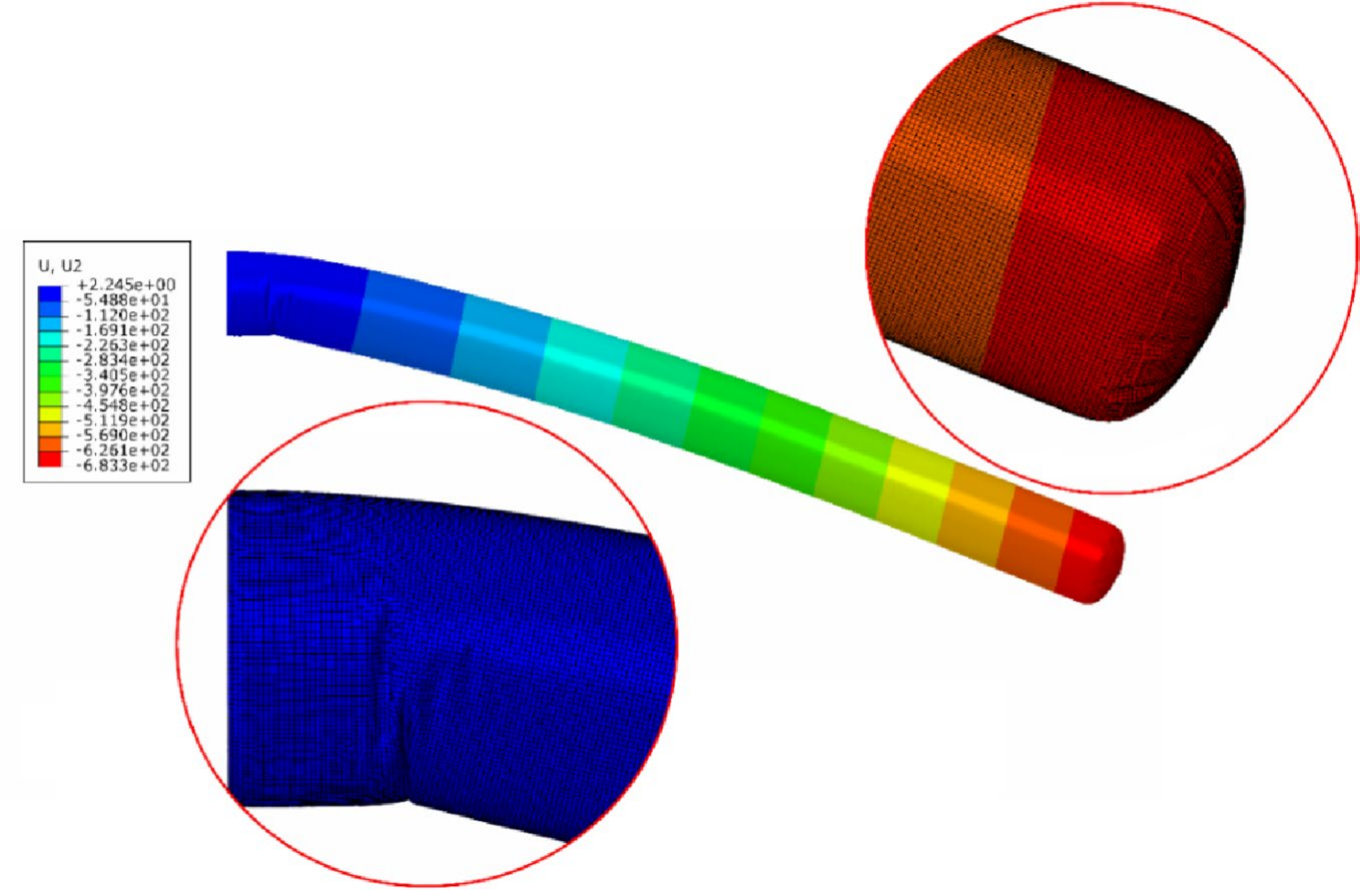
Wrinkling of the cantilever beam with 155 N applied force at the free end (250 mm diameter beam at 1 bar inflation pressure).

The results showed that the average error for the deflection before wrinkling was 0.88% and the maximum error for all the cases is 4.76%. These minor errors within 5% may be attributed to material thickness irregularities especially at the welded joints. And it is observed from the curves in (Fig. 38) that increasing pressure makes the beam more hyperelastic than the linear acting beam. (Fig. 39) shows the response of the 250 mm beam inflated at 1 bar and subjected to a 155 N force at its end. The developed model was able to capture the appearance of wrinkled membranes occurring at the beam root and tip. The simulation model shows that the wrinkling phenomenon dramatically decreases the stiffness of the beam making it difficult to maintain the stability of the structure.

**Fig. 40:**
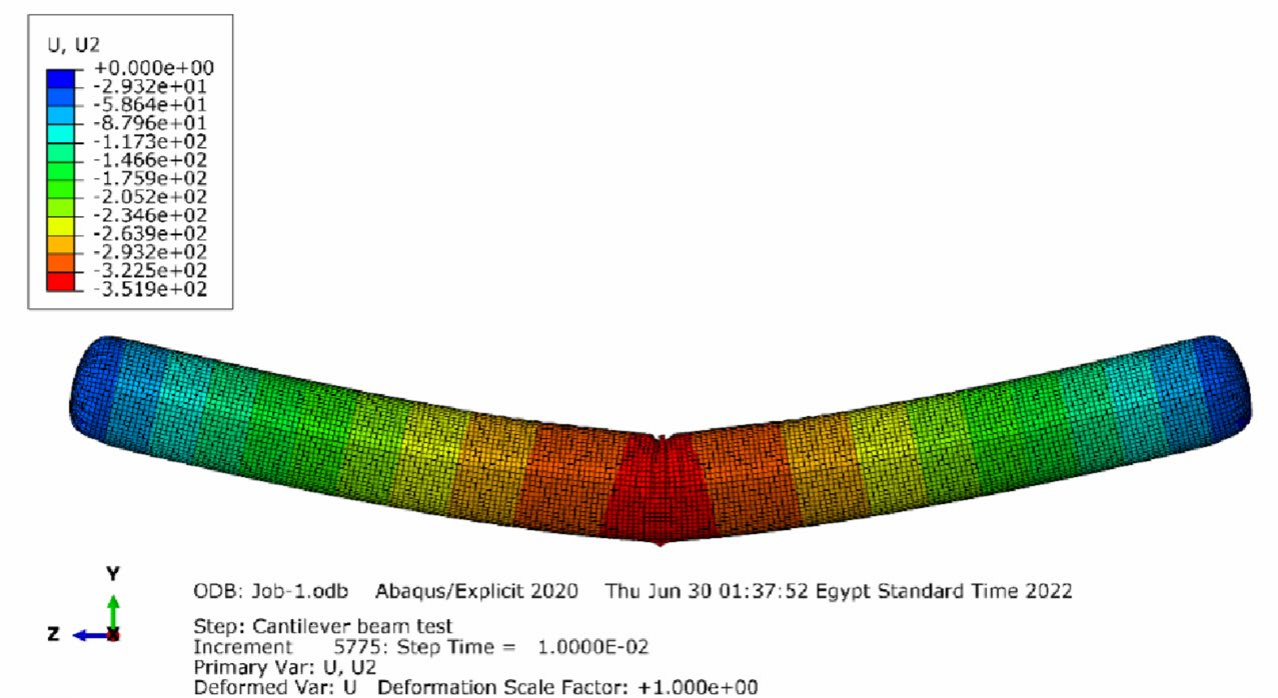
Deflection of 300 mm diameter beam (simply supported) under external force of 450 N, where wrinkling phenomenon is clearly observed; units are in [mm].

More simulation cases have been tested using the developed framework including an inflated beam simply supported at its ends. (Fig. 40) shows the response of a 300 mm diameter beam inflated at 1.5 bar subjected to a 450 N load in the middle of the beam.

**Fig. 41:**
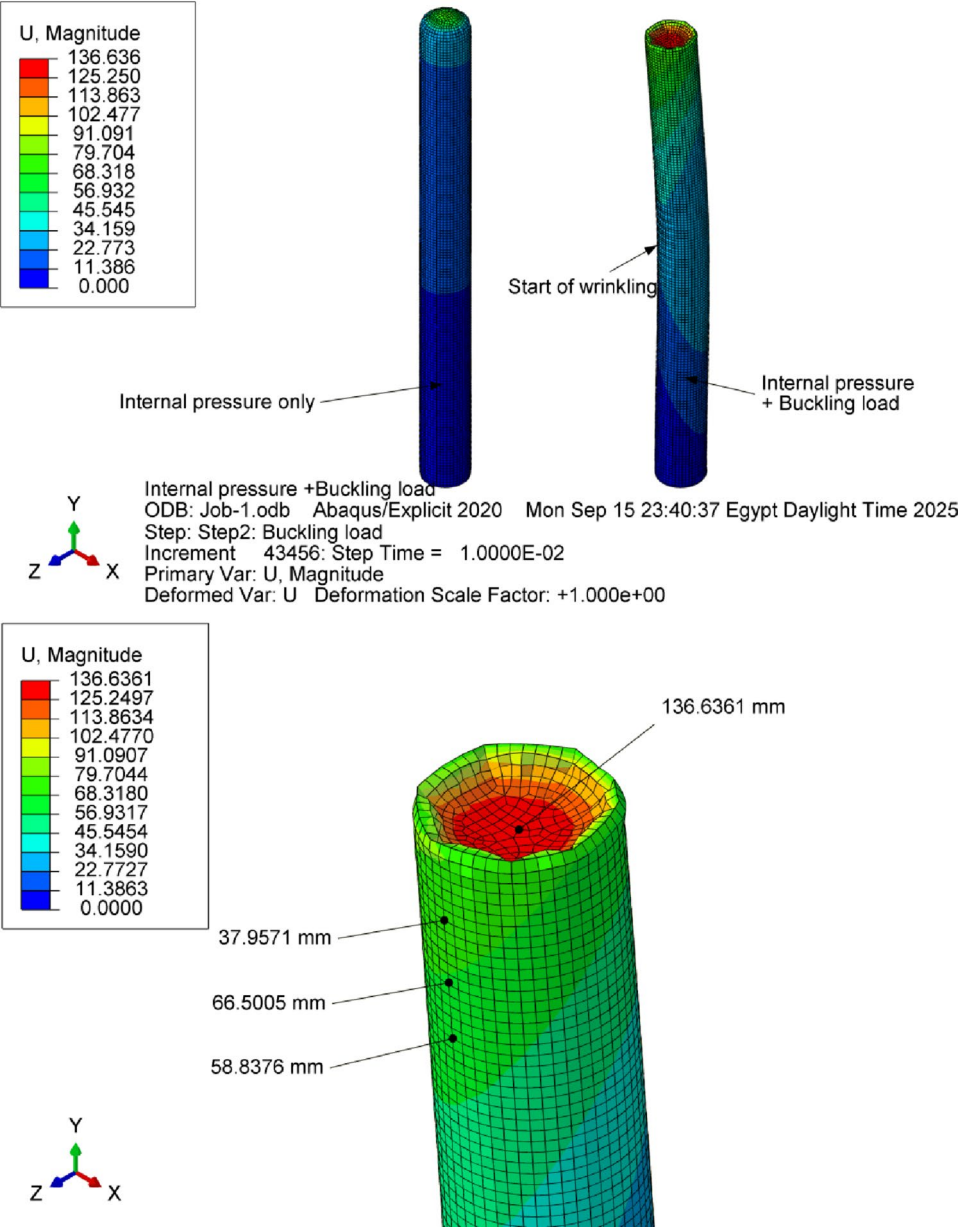
Behavior of the inflated beam (300 mm diameter–3300 mm long) under compression of 33 N.

The structural integrity of the developed model was further evaluated through buckling analysis by applying axial loads to the inflated beam with 1.5 bar inflation pressure (fixed-free loading case). (Fig. 41) shows the beam suffers axial wrinkling when subjected to a compressive load of 33 N, highlighting the significant sensitivity of those inflatable beams to axial loading conditions.

It can be concluded from this section that the model is capable of accurately predicting the behavior of the straight simple or cantilever supported beam under different loads, the increase of the beam stiffness with the increase of the internal pressure or beam diameter, and the nonlinear behavior of the beam including local buckling and wrinkling of the membrane under specific loading conditions. Hence, the model is reliable and can be used in structural investigation of more free-form inflatable structures.

## 4. Discussion

### 4.1. Rationale behind the chosen test cases

The results of the test cases presented herein demonstrate the robustness of the modeling approach in addressing manufacturable inflatable fabrics with real constraints. Table 5 presents a summary of the modeling results, together with a comparison against 3D photogrammetry scans for validation purposes. These results indicate that the FEA model provides a reliable prediction of the inflated free-form shapes.

The inflated pillow serves as a fundamental baseline case for the validation of material behavior, seam modeling, and geometric accuracy without introducing additional structural complexity. Manufacturing defects, including welding imperfections, are recognized as dominant factors influencing the inflatables’ geometric response. Rather than treating these imperfections as sources of error, the proposed model can be employed to investigate their effects on structural performance by introducing small deviations in CZM modeling parameters such as seam width, normal and shear stiffness, or cohesive strength. This allows the framework to quantify sensitivity to manufacturing imperfections, enabling designers to establish acceptable fabrication tolerances before prototyping. The inflated cuboid with internal stiffeners introduces internal constraints and load path complexities, making it ideal for evaluating the interaction between reinforcements and the fabric, as well as capturing localized wrinkling or buckling. The twisted lofted geometry represents a highly asymmetric, free-form structure, challenging the simulation to capture large deformations, twist-induced instabilities, and geometric nonlinearity, which are from the key aspects in real-world applications such as architectural membranes. Finally, the twisted lofted geometry with internal stiffeners combines all the previous complexities, providing a critical test for the ability of the framework to handle the coupled effects of torsion, reinforcement, and material anisotropy, making it an advantageous case for validation, as summarized in (Table 5).

**Table 5:** Comparison of the four test cases.

Test case	FEA model elements	Purpose	Behavior	Deviation	Key issues
1. Symmetric Pillow (2D-pillow)	21,976	Material + seam behavior	Localized deformations, wrinkling at welded seams	± 13 mm (lateral), ± 7 mm (transverse)	Corner twirl effect not predicted, welding defects impacting the results
2. Symmetric Cuboid with inner Stiffener	181,655	Load distribution + welds	More uniform stress distribution, less localized deformation	± 6.2 mm	Model predicted geometry with high accuracy, minimal error
3. Lofted Twisted Structure	230,470	Free-form geometry	Reduction in twist angle, nonlinear relaxation behavior	Twist angle: changed from 20° to 15°	Difficulty capturing complex stress redistribution requires further pressure range analysis
4. Lofted Twisted Structure with Stiffeners	287,890	Combined behavior	Stiffeners reduce twist, optimize stress distribution	Twist angle changed from 20° to 16° (reduced effect)	Ineffective diagonal stiffener placement, suggesting optimization needed for best stiffener configuration

#### 4.1.1. [2D] symmetric pillow

For the symmetric pillow model, the FEA predicted the inflated shape with a maximum deviation of ± 13 mm in the lateral cross-section and ± 7 mm in the transverse cross-section. These discrepancies are attributed to welding defects, as the measured peel normal strength of the welded joint is 10 MPa (see Sect. 2.3). And with a weld width of 30 mm, this strength provides a conservative margin of safety under the applied internal pressure (1.5 bar). Therefore, the observed irregularities are primarily the result of poor or inconsistent welding quality rather than insufficient predictive ability of the current FEA model. In addition, the corner twirl observed in the 3D scan – but absent in the simulation – highlights the need to enhance the current FEA modeling technique to better capture such localized phenomena.

#### 4.1.2. [3D] symmetric cuboid with inner stiffener

The cuboid model exhibited a maximum geometrical error of ± 6.2 mm, aligning closely with the FEA predictions. This consistency suggests that the current modeling approach is effective for structures with internal stiffeners, where the stress distribution is more uniform and less deformation occurs.

#### 4.1.3. [3D] asymmetric lofted twisted structure

The lofted twisted structure presented challenges in predicting the twist angle, which decreased from 20° to 15° under inflation. This reduction reflects the structure’s evolution toward an equilibrium configuration governed by internal pressure and membrane mechanics (strain energy in the fabric). In inflatable membranes, pressure acts normal to the surface, driving deformation toward smooth, doubly curved, force-balanced forms (cylinder or sphere).

The initially imposed twist introduces geometric asymmetry and nonuniform curvature, generating nonuniform membrane stresses. Upon pressurization, the structure tends to minimize its total potential energy – comprising both the strain energy stored in the membrane and the work done by pressure – by deforming toward a more symmetric, energy balanced shape. This relaxation partially reduces the twist, enabling a more efficient distribution of stresses and surface normal. This behavior is consistent with established principles in the mechanics of thin membranes and pressure-driven equilibrium geometry, as discussed by Selvadurai^37^ and underscores both the influence of internal pressure and the complexity of modeling such geometries. Further studies across broader pressure ranges are recommended to better characterize this relationship.

The introduction of this twist relaxation behavior for inflatables is essential for different engineering applications, especially those with twisted geometries or sections like wind turbine blades, where maintaining a designed twist distribution is essential for optimal aerodynamic performance. Relaxation of the twist after inflation or under aerodynamic loading can reduce the effective angle of attack along the span, leading to nonuniform lift distribution and a decrease in the overall efficiency. This effect suggests that a controlled pre-twist be introduced during design to compensate for the expected relaxation once the structure is inflated or loaded. Furthermore, the predictive capability of the proposed FEA framework allows designers to simulate this behavior in advance, enabling optimization of geometric pre-form to achieve the target twist under operational pressure.

#### 4.1.4. [3D] asymmetric lofted twisted structure with inner stiffeners

Introducing inner stiffeners to the lofted twisted structure reduced the initial twist angle from 20° to 16°. The stiffeners’ configuration strongly influenced the deformation pattern, highlighting the importance of strategic placement. The analysis revealed that diagonal arrangements were less effective, resulting in greater deformation, whereas aligned stiffeners provided improved structural integrity. Discrepancies observed near weld lines are likely due to simplified seam modeling and material property variations not captured by uniaxial tensile tests. In addition, localized wrinkling observed experimentally may not be fully represented in the simulation because of mesh coarseness. Overall, this case demonstrates the framework’s ability to capture key stiffener-membrane interactions while also indicating areas where seam modeling and mesh refinement require further development.

### 4.2. Model validation for external loading

The proposed framework was further validated for its ability to predict the behavior of inflatable structures under external loading. The first level of validation involved comparison with experimental results from compression tests on inflated free-form structures, while the second level was based on data previously collected on an inflatable cantilever beam.

The framework predicted a geometric stiffness of 15.78 N/mm for case 3 and 47.07 N/mm for case 4, compared to measured values of 14.19 N/mm and 44.83 N/mm, respectively. These deviations are likely due to the presence of the pressure regulator, which maintains constant internal pressure during testing, whereas in the simulation the pressure increases with compression. This suggests that a potential modification linking the pressure to the movable compressing jaw in the FEA model that could improve predictive accuracy. Overall, the close agreement between predicted and measured values confirms the framework’s reliability in capturing both global stiffness and load–deflection behavior of complex free-form inflatable geometries.

For the data from Okda et al.^36^, in which cantilever inflatable beams were tested under various loads and inflation pressures. The FEA model demonstrated an average error of 0.88% in the deflection predictions, with a maximum deviation of 4.76%. These minor discrepancies are likely due to material thickness variations and welding inconsistencies. The simulation successfully captured the nonlinear behavior of the beams, including the onset of wrinkling under specific loading conditions. The appearance of wrinkling significantly reduced the beam stiffness, aligning with the experimental observations. This capability underscores the model’s potential for analyzing complex inflatable structures under diverse loading scenarios.

### 4.3. Implications for design

The findings from this study have several implications for the design and analysis of inflatable structures such as accurate orthotropic material representation with plasticity effect using VUMAT subroutine. This study highlights the importance of optimizing stiffener configurations to achieve the desired geometry. The observed discrepancies in corner regions and twist behavior suggest areas for improvement in the FEA model while incorporating advanced contact models and material nonlinearities could enhance prediction accuracy.

## 5. Conclusions

This study presents a finite element analysis (FEA) framework capable of predicting the final inflated geometry of free-form inflatable structures, including unconventional designs with non-tubular chambers or chamber-less configurations. The framework integrates full material characterization of PVC-coated fabrics into a computational model implemented in Abaqus, addressing critical challenges such as large deformations (wrinkling, buckling), anisotropic material behavior, fluid-structure interaction, and weld seam uncertainties.

The framework was rigorously validated in two phases: the first phase is geometric accuracy – comparisons with 3D photogrammetry scans of four representative structures (double-layer pillow, stiffened cuboid, twisted lofted form, and its stiffened variant) demonstrated reliable prediction of final inflated shapes, with maximum deviations of ± 13 mm in lateral cross-sections and ± 7 mm in transverse sections. These test cases specifically addressed knowledge gaps in weld anisotropy, geometric nonlinearity in asymmetric forms, and load-path complexities from internal stiffeners.

The second phase is mechanical performance – validation against analytical solutions for inflated beams confirmed high accuracy in capturing nonlinear stiffness variation and wrinkling, with average errors below 5% in deflection predictions.

The ability of the framework to simulate complex geometries – such as cuboids and twisted forms – highlights its transformative potential for applications ranging from architectural membranes to renewable energy systems. Notably, it has enabled the design of inflatable wind turbine blades (patents: EG/P/2024/1240 and PCT/EG2025/050007^20^) by maintaining aerodynamic precision under operational loads.

### Limitations and future work

The proposed framework significantly advances the predictive modeling of inflatable structures, capturing both geometric and mechanical behavior with high fidelity. Nonetheless, several intrinsic limitations remain, highlighting opportunities for further development. Fatigue life under cyclic loading remains unaddressed, representing a critical gap for applications such as inflatable wind turbine blades. Viscoelastic phenomena, including long-term creep and stress relaxation at welded seams, require quantification to ensure structural durability. Current element formulations and joint models are insufficient for accurately capturing edge and corner behavior, and anisotropy evolution due to fiber reorientation at strains exceeding 15% demands further VUMAT enhancements. Moreover, systematic sensitivity analyses are essential to evaluate how inflation pressure, material properties, stiffener configurations, twist angles, and boundary conditions influence final geometry and stiffness, guiding informed design decisions. Despite these limitations, this study establishes a foundational tool for designers, offering immediate applicability in energy and aerospace systems, while setting the stage for future extensions to dynamic loading, material degradation, and robust real-world performance predictions.

## Supplementary Information

Below is the link to the electronic supplementary material.

Supplementary Material 1

## Data Availability

Most of the data supporting the findings of this study (including generated or reused data) are available to the public and can be accessed via the following link: https://engasuedu-my.sharepoint.com/:f:/g/personal/amir_azer_eng_asu_edu_eg/Ekp4GfdWHZJDr1PNLgQvofYB9tRlQye9WImpYVeMLuBADg? e=JhHe3xTension test data are publicly available in the zenodo repository, with DOI: [https://doi.org/10.5281/zenodo.14232076](https:/doi.org/10.5281/zenodo.14232076)Any further required data are available from the corresponding author upon request.
