# Supplementary material for: A numerical simulation approach for inflatable asymmetric geometries of orthotropic fabrics
Source: Sci Rep. 2026 Mar 10;16:8596. doi: 10.1038/s41598-026-40016-5 (PMC12976094; doi:10.1038/s41598-026-40016-5)
Supplement: Supplementary file 1 — Supplementary Material 1 [file 41598_2026_40016_MOESM1_ESM.pdf]

Supplementary material of the manuscript entitled:

A numerical simulation approach for inflatable asymmetric geometries of orthotropic fabrics

Amir Samir Azer Abdelmaseeh [1, *], Adel Elsabbagh [2, 4, +] Amr Yehia Elbanhawy [3, 5, +]

[1] Teaching assistant, Mechanical Design and Production Department, Faculty of Engineering, Ain Shams University, Cairo, 11517, Egypt

[2] Professor, Mechanical Design and Production Department, Faculty of Engineering, Ain Shams University, Cairo, 11517, Egypt

[3] Associate Professor, Mechanical Power Department, Faculty of Engineering, Ain Shams University, Cairo, 11517, Egypt

[4] Center for Vibration, Sound and Smart Structures, Faculty of Engineering, Ain Shams University, Cairo, 11517, Egypt

[5] Energy Technology and Climate Change Laboratory, Faculty of Engineering, Ain Shams University, Cairo, 11517, Egypt

Corresponding author: Amir Samir Azer Abdelmaseeh (amir.azer@eng.asu.edu.eg)

1. Supplementary Note 1 – Detailed geometric analysis of the [2D] Symmetric pillow

The finite element model comprised 21,976 elements. For 3D scanning via photogrammetry, 227 photographs were captured, generating a point cloud of 322,000 points. The initial surface reconstruction yielded 10.4 million faces, which were subsequently optimized to 400,000 polygons to balance computational efficiency and geometric fidelity.

The welding defects (peel adhesion irregularities) affecting the gematrical deviations in the lateral cross section are shown in (Supplementary Figure 1), and the corner twirl effect observed during inflation and not captured by the FEA model is shown in (Supplementary Figure 2).

The quantitative deviation errors between the 3D-scanned geometry and the numerical predictions are measured to be between [-13.79, +1.31] mm for the lateral cross-section (Supplementary Figure 3) and [-7.2, +5.68] mm in the transverse cross-section (Supplementary Figure 4).

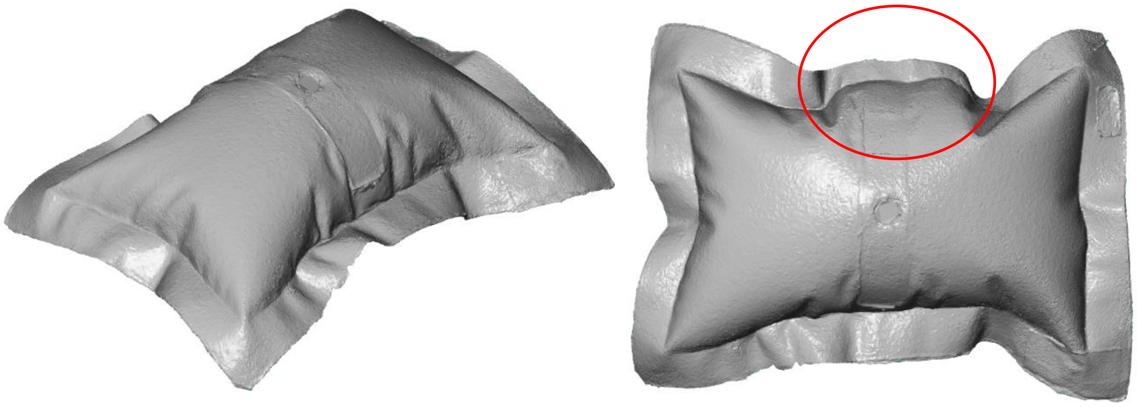

Supplementary Figure 1 [3D] scanned inflated model of the manufactured part (2D membrane pillow). The weld is corrupted at the upper part of the test sample.

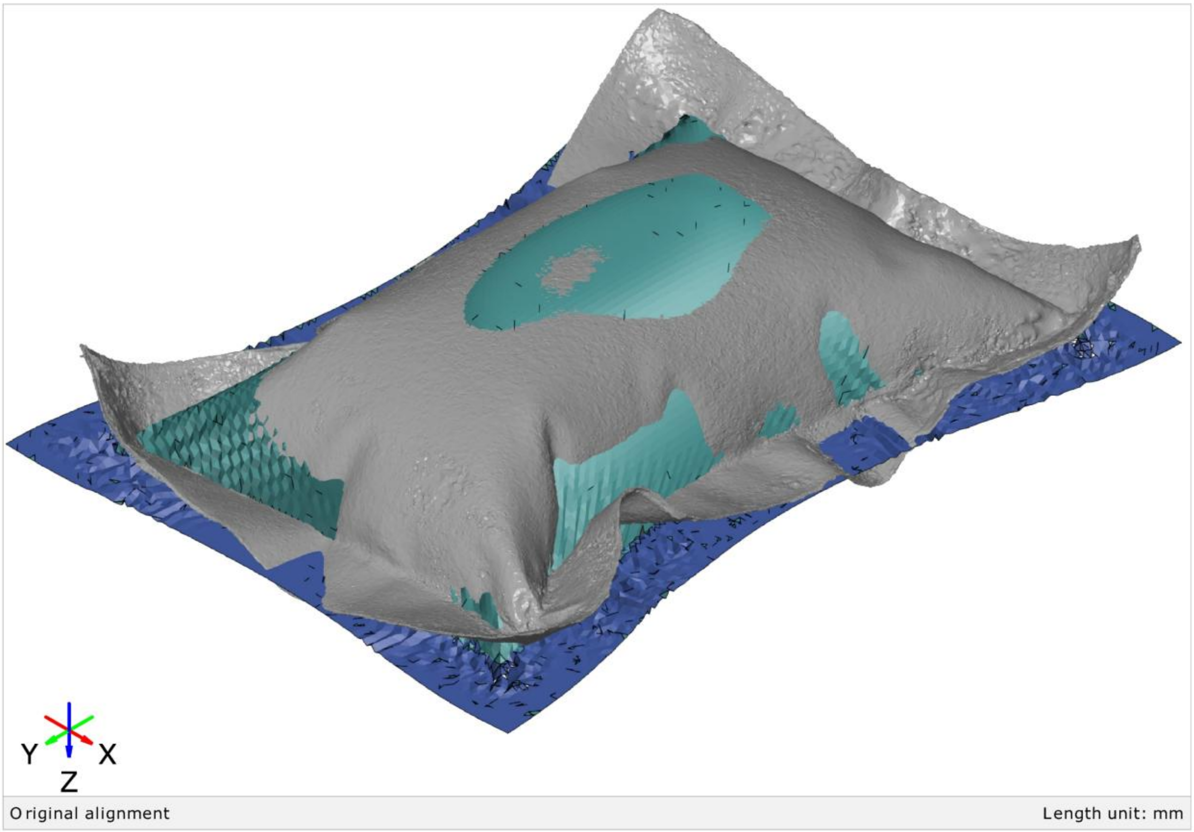

Supplementary Figure 2 Scanned geometry (gray) versus FEA simulations (blue). Free edge (corner twirl) effect that does not appear in the simulation.

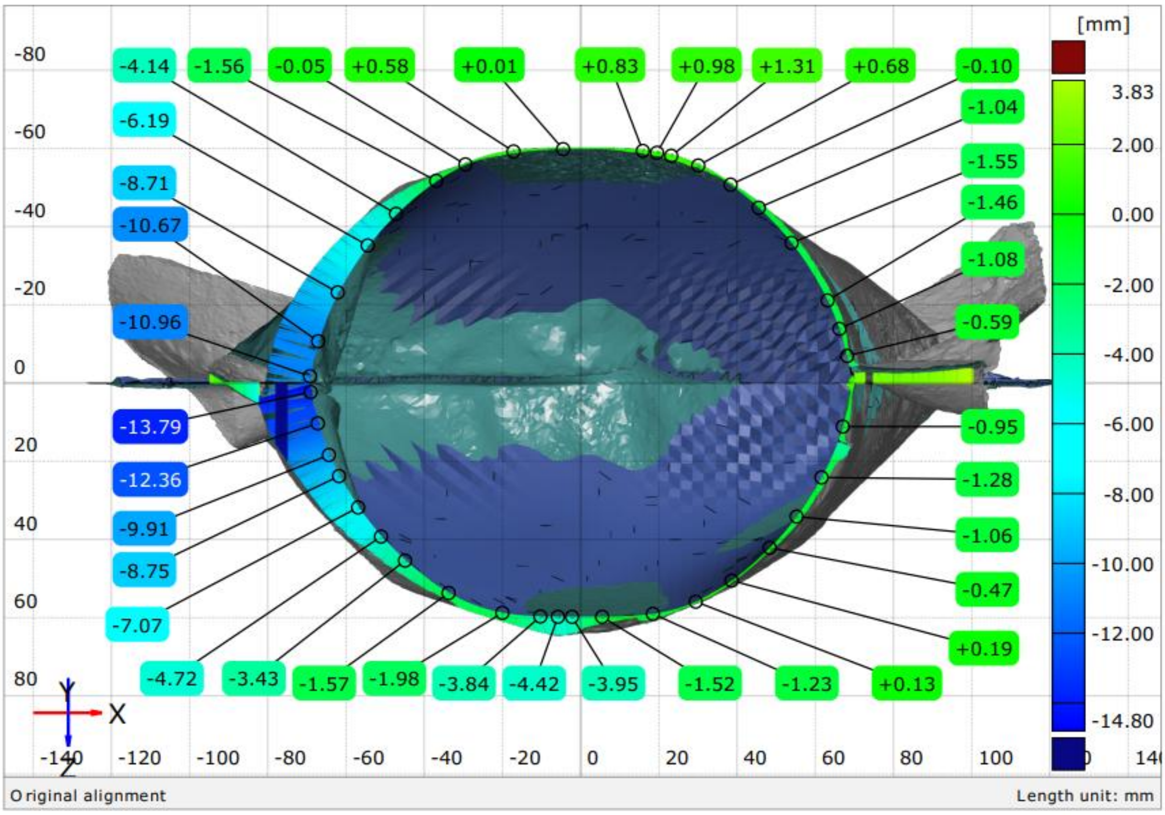

Supplementary Figure 3 Lateral cross- sectional geometrical error of the 3D scanned inflatable 2D pillow at (1.5 bar) pressure compared with the model predicted via [GOM Inspect] software (ZEISS).

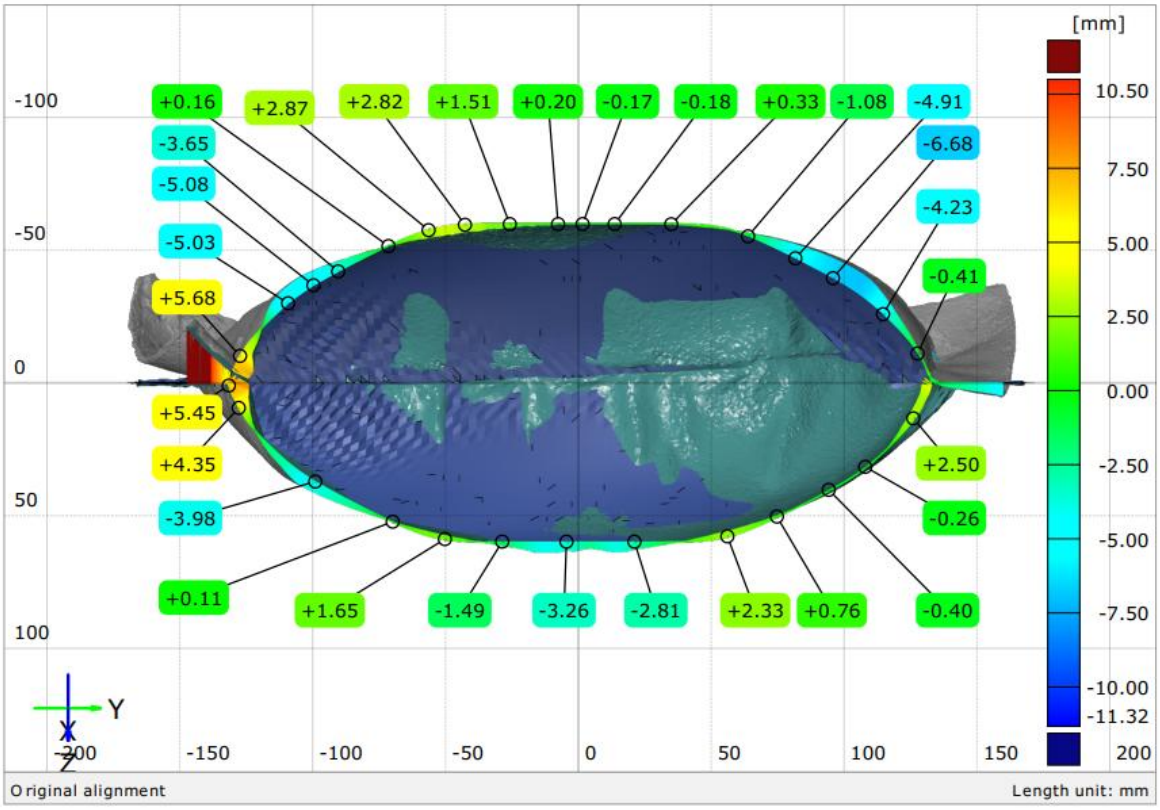

Supplementary Figure 4 Transverse cross-sectional geometrical error of the 3D scanned inflatable 2D pillow at (1.5 bar) pressure compared with the model predicted via [GOM Inspect] software.

2. Supplementary Note 2 – Detailed geometric analysis of the [3D] cuboid with inner stiffener

The FEA model consisted of 181,655 elements, while 3D scanning photogrammetry utilized 230 photographs to generate a point cloud of 459,000 points (Supplementary Figure 5). The initial mesh reconstruction produced 10 million faces, which were later optimized to 400,000 polygons. The geometrical deviation between the 3D scanned inflatable cuboid, and the numerically predicted model (evaluated via GOM Inspect software) is ranging between [-6.21, +2.94] mm across the transverse section (Supplementary Figure 6).

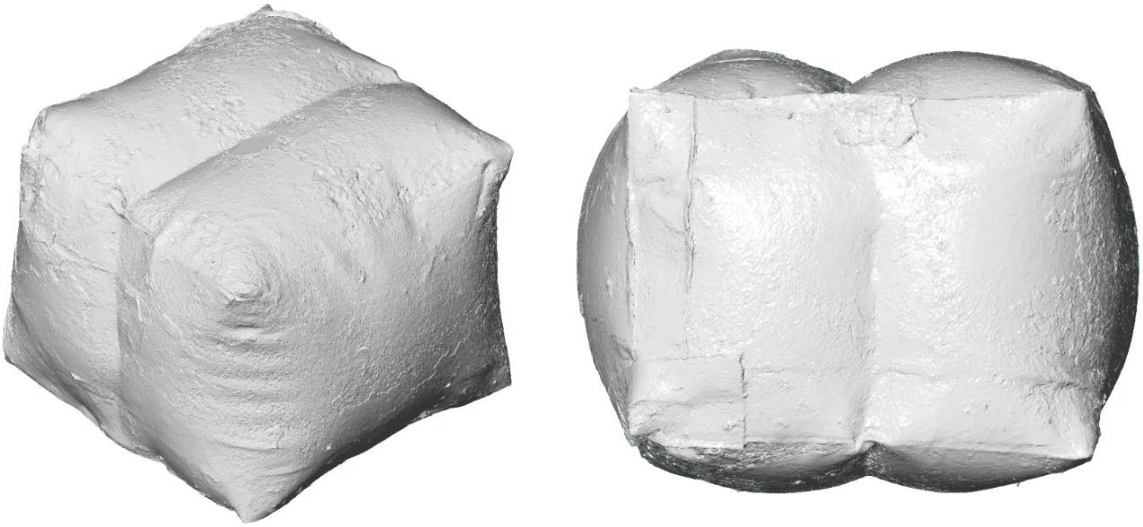

Supplementary Figure 5 [3D] scanned inflated model of the manufactured part (cuboid shape with middle stiffener).

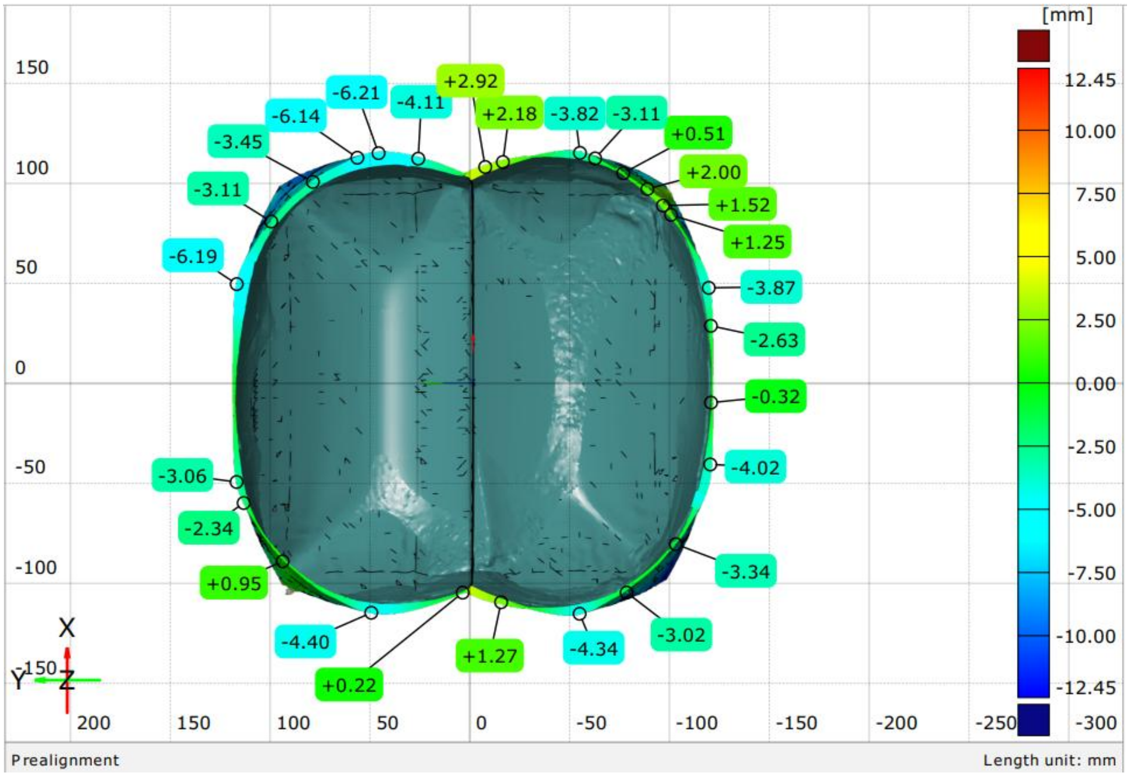

Supplementary Figure 6 Geometrical error of the 3D scanned inflatable cuboid, compared with the model predicted via [GOM Inspect] software.
